# Local anaesthetic to reduce injection pain in patients who are prescribed intramuscular benzathine penicillin G: a systematic review and meta-analysis

**DOI:** 10.1016/j.eclinm.2024.102817

**Published:** 2024-09-04

**Authors:** Ferruccio Pelone, Bessie Kwok, Sabahat Ahmed, Yakup Kilic, Syed Ahsan Ali, Nida Ahmed, Mahmood Ahmad, Jonathan JH. Bray, Farhad Shokraneh, Miryan Cassandra, David S. Celermajer, Eloi Marijon, Rui Providencia

**Affiliations:** a Institute of Health Informatics Research, University College London, London, UK; b Barts Heart Centre, St Bartholomew's Hospital, Barts Health NHS Trust, London, UK; c University College Hospital, University College London Hospitals NHS Trust, London, UK; d Cardiology Department, Royal Free Hospital, Royal Free London NHS Foundation Trust, London, UK; e Oxford Heart Centre, John Radcliffe Hospital, Oxford, UK; f Cardiology Department, Hospital Dr. Ayres de Menezes, São Tomé, São Tomé e Príncipe; g Faculty of Medicine and Health, The University of Sydney, Sydney, Australia; h Paris Cardiovascular Research Centre, INSERM U970, European Georges Pompidou Hospital, Paris, France

**Keywords:** Lidocaine, Rheumatic, Syphilis, Impetigo, Streptococcal pharyngitis

## Abstract

**Background:**

Three to 4-weekly intramuscular injections of benzathine penicillin G (BPG) for a prolonged period (e.g., 10 years, until age 40 years, or lifelong) are recommended for preventing group A streptococcal infections that cause recurrent acute rheumatic fever (ARF) and potential progression to rheumatic heart disease (RHD). The duration of treatment, frequency and local pain associated with BPG injections may lead to reduced compliance. Shorter courses of BPG are recommended for the treatment of syphilis and Streptococcal infections. We aimed to assess the effects of local anaesthesia in reducing injection pain in patients who are being treated with BPG.

**Methods:**

In this systematic review and meta-analysis, we searched the Cochrane Central Register of Controlled Trials, MEDLINE, EMBASE, Conference Proceedings Citation Index-Science and LILACS from database inception up to May 4, 2024, and performed additional searches for grey literature. Randomised controlled trials comparing BPG vs. BPG administered alongside local anaesthetics were included. Randomized controlled trials using BPG, irrespectively of indication, and testing any local anaesthetic agent for pain alleviation were considered eligible. We applied GRADE to assess the quality of evidence. Summary data were extracted from included trials. The primary outcome was injection pain, assessed through mean differences. A random-effects model was utilized to account for study heterogeneity. This study is registered with PROSPERO, CRD42022342437.

**Findings:**

Database searches identified a total of 3958 records, and 3 additional records were retrieved from grey literature searches. After removal of duplicates, screening of abstracts and full-text review, eight trials were included, combining a total of 489 patients (151 patients with RHD). Immediate pain level, as reported by patients, was of high intensity in most studies. Low intensity pain was still reported at 24 h. Administration of lidocaine mixed with BPG was associated with a significant reduction in immediate post-injection pain (mean difference −3.84, 95% confidence interval −6.19 to −1.48, P = 0.0001; 4 studies; I^2^ = 98%; GRADE: moderate quality), pain at 5 min (mean difference −2.85, 95% CI confidence interval −3.78 to −1.92, P < 0.0001; 1 study; GRADE: moderate quality), and pain at 20 min (mean difference −1.85, 95% confidence interval −2.61 to −1.09, P < 0.0001; 1 study; GRADE: moderate quality) on a 1 to 10 scale. One study assessed lidocaine cream applied to the skin prior to BPG injection and showed no significant reduction in injection pain (mean difference = −0.54, 95% CI confidence interval −1.17 to 0.09, P = 0.13; 1 study; GRADE: low quality). Mepivacaine mixed with BPG in patients with syphilis showed a significant reduction of immediate post-injection pain (mean difference −2.19, 95% CI confidence interval −2.49 to −1.89, P < 0.0001; 1 study; GRADE: moderate quality). Two studies assessed procaine mixed with BPG and reported: lower immediate pain levels or pain assessed at 1 h (mean difference and 95% CI confidence intervals not provided, P = 0.001 and P = 0.008, respectively; 1 study; GRADE: low quality), or less immediate pain and pain at 24 h on the buttock injected with procaine mixed with BPG (mean difference and 95% CI confidence intervals not provided, P < 0.001 for both; 1 study; Grade: low quality). No severe adverse reactions were reported.

**Interpretation:**

In patients receiving intramuscular BPG injections, moderate quality quantitative evidence suggests that BPG injections diluted with lidocaine or mepivacaine may improve post-injection pain scores compared to BPG injections diluted with sterile water. Procaine may also have a benefit, but quality of evidence was lower. Most studies included small patient samples and assessed pain levels at different timepoints. Due to insufficient data we were not able to assess the impact of injection volume, and local anaesthetics’ dose on pain intensity and duration of pain relief.

**Funding:**

10.13039/100004423WHO.

**Research in context**

**Evidence before this study**

Preliminary searches on MEDLINE, EMBASE, Conference Proceedings Citation Index-Science and LILACS from inception to April 2023, and repeated on May 4th 2024, without language restrictions and using the terms “pain”, “penicillin”, “local anaesthetics”, “systematic review” and “trial”, identified no prior systematic reviews on the safety and efficacy of local anaesthetics combined with intramuscular BPG.

**Added value of this study**

This is the first systematic review on local anaesthetics for pain management in patients treated with BPG, providing the largest available body of randomised controlled trial evidence on the topic (8 randomized controlled trials combining a total of 489 patients, 151 with rheumatic disease). No severe adverse reactions were reported. Evidence of moderate quality suggests that lidocaine mixed with BPG causes moderate to major clinically meaningful reduction in immediate, 5 min and 20 min post-injection pain. Similarly, mepivacaine mixed with BPG in patients with syphilis significantly reduces immediate post-injection pain. Evidence of low quality suggests that: lidocaine cream applied to the skin prior to BPG injection does not reduce injection pain, and procaine mixed with BPG may significantly lower immediate and 1 h pain levels.

**Implications of all the available evidence**

Utilisation of local anaesthetics appears to be safe and may reduce immediate post-injection pain in individuals requiring treatment with BPG. Safety data is scarce, but reassuring based on the utilized low local anaesthetic doses. Further research on the use of subcutaneous BPG with lidocaine, and combination of local anaesthetics with other pain alleviating strategies is warranted.

## Introduction

Acute rheumatic fever (ARF) is an auto-immune inflammatory condition occurring as a result of an upper respiratory tract infection caused by group A beta-haemolytic streptococci. ARF typically occurs 10–21 days after the infection and may affect the heart, and eventually lead to long-term structural and functional change in the heart muscles and valves (Rheumatic Heart Disease—RHD).^1^

Secondary prevention with antibiotics is vital to prevent further episodes of ARF and potential development of RHD.^2^ Continuous administration of antibiotics to patients with a previous episode of ARF or already existing RHD is therefore recommended.^3^ Three to four-weekly intramuscular benzathine penicillin G (BPG) for a prolonged period (e.g., 10 years, until age 40 years, lifelong) has been demonstrated to be the most effective antibiotic for this purpose and is superior to oral chemoprophylaxis.^4^^,^^5^ A compliance rate of 80% of prescribed BPG injections is required to significantly reduce the risk of RF recurrence.^6^ Maintaining this degree of adherence remains a challenge worldwide for a variety of reasons such as difficulty attending clinics every 3–4 weeks for a prolonged period of time, financial constraints and the pain of injections.^7^

BPG is also indicated for the treatment of syphilis, with a single BPG injection recommended for early stages (primary, secondary and early latent syphilis), and repeated injections recommended for late latent and tertiary syphilis (three doses at 1-week intervals).^8^^,^^9^ More detailed information on posology for the aforementioned indications, and for Streptococcal infections are provided in Appendix 1.

Pain due to intramuscular injection of BPG is described as severe, with >50% in a cohort of 100 children describing it as an 8 or 10 in a scale of 0–10.^10^ Consequently, there is a need to identify effective methods to reducing this pain to improve adherence to secondary prevention measures for RHD and ARF.^11^ Strategies aimed at managing pain, fear, and distress of benzathine penicillin G injections can include different options including local anaesthetics mixed with the injection and other methods.^12^ When a local anaesthetic agent is added to BPG it may reduce the pain of injection, without significantly affecting serum BPG concentrations in serum.^10^^,^^12^ However, local anaesthetics have potential for side effects such as allergic reactions, neurologic reactions (e.g., seizures), or other adverse reactions.^13^ Accordingly, the World Health Organization (WHO) guideline development group (GDG) for the Clinical practice guidelines on the prevention and management of ARF and RHD felt an evidence synthesis process was required to address the question: *“Should patients who are prescribed intramuscular BPG receive a local anaesthetic to reduce injection pain?”*. During the development of the Guideline, due to the scarce number of trials in the ARF/RHD population, it was suggested that the aim of the review should extend beyond ARF/RHD and assess the efficacy and safety of local anaesthetics to reduce injection pain in all patients prescribed with BPG (any treatment indication).

## Methods

This systematic review adhered to the PRISMA guidelines^14^ and the protocol was registered and published online on PROSPERO.^15^

### Search strategy and selection criteria

We searched the following sources relevant studies from database inception up to 04/05/2024: CENTRAL (Cochrane Central Register of Controlled Trials), MEDLINE (Ovid), Embase (Ovid), Conference Proceedings Citation Index-Science (CPCI–S) (Web of Science), and LILACS (Literatura Latino-Americana em Ciências da Saúde).

Each source was scanned by means of a specific search string combining a database-specific mix of subject headings and text words (summarized in Appendix 1). Searches were deliberately broad with the aim of identifying all trials of patients prescribed with BPG and local anaesthetics. Additional searches for grey literature were done on Google using the same combination of text words.

We utilized the PICO approach. The population included patients prescribed intramuscular BPG. Initially, studies were restricted to secondary prevention of patients with RHD, but on advice from the WHO guideline development group (GDG) this was expanded to any patients being treated with intramuscular BPG as pain-related considerations and effect of local anaesthetics were considered to be the same. The intervention was utilization of any local anaesthetic with the aim of reducing injection pain. Anaesthetics of interest included: Amylocaine, Benzocaine, Bupivacaine, Chloroprocaine, Etidocaine, Lidocaine, Oxybuprocaine, Mepivacaine, Prilocaine, Procaine, Proparacaine, and Tetracaine. The comparators included placebo or other interventions for reducing injection pain. Types of study eligible for this review were randomised controlled Trials (RCTs), including those using a cross-over methodology.

Studies were excluded because they did not meet the inclusion criteria or because the met the following exclusion criteria: any type of literature review (e.g., narrative, systematic, meta-analysis, and meta-synthesis), although they were used as sources of potentially relevant studies; case reports and case series, because of the limited possibility of generalizing the validity of these studies^16^^,^^17^; conference abstracts, unless there is enough detail for data extraction; longitudinal and cross-sectional survey research, because of the limited possibility of establishing a cause-effect relationship of these non-comparative studies^16^; papers not reporting empirical findings (e.g., editorials, comments with no primary data); and studies reporting on antibiotics other than intramuscular BPG. There were no language restrictions.

We measured the outcomes at all available time points. Injection pain was the primary endpoint and was measured as a “pain score” or other type of reporting used in the investigation. The secondary endpoints included: fear (measured through a questionnaire^18^ or other method reported by the authors), adherence to treatment,^19^ acceptability to provider and patient (measured as patient and provider preference) and serious and non-serious adverse events (any).

All references identified by the searches and from other sources were uploaded into Rayyan Systematic Review software and de-duplicated. Titles and abstracts of the retrieved citations were screened to identify studies that potentially meet the inclusion criteria outlined in the review protocol. Dual screening was performed on all records identified by the search (FP, BK, SA, YK, RP); 90% agreement was required. Disagreements were resolved via discussion between the two reviewers, and consultation with a third reviewer if necessary (MA). Full versions of the selected studies were obtained for assessment. Studies failing to meet the inclusion criteria once the full version had been checked were excluded.

Data extraction consisted of the following: study design, number of participants, male/female, age, ethnicity, country, indication for BPG, type of housing, rural area vs. urban, form, dosage and type of local anaesthetic drugs, previous ARF, history of RHD, World Heart Federation (WHF) criteria, RHD diagnosis data, syphilis stage, comorbidities (e.g., HIV), dose and frequency of BPG administration (every 3 weeks, 4 weeks, other), length of antibiotic course, needle gauge, study follow-up duration, outcomes data and definition.

For each included study, two reviewers in parallel extract relevant data into a standardised form. This was quality assessed by a third reviewer. Specifically, four review authors (FP, BK, SA, YK) individually and performed a dual data extraction using standardised form was used to extract data from studies, designed specifically for this synthesis. Discrepancies between review authors were resolved by peer discussion.

### Data analysis

The Cochrane Risk of Bias (RoB) tool for RCTs^20^ was used to assess risk of bias/methodological quality of included papers. The quality assessment was performed independently by four reviewers (FP, BK, SA, YK), and this was quality assessed by a third reviewer (MA or RP).

We applied the five GRADE considerations (study limitations, consistency of effect, imprecision, indirectness and publication bias) to assess the certainty of the body of evidence as it relates to the studies which contribute data to the meta-analyses for the prespecified outcomes.^21^ The overall ‘Risk of bias' judgement for each study was used as part of GRADE assessment.^22^ We justified all decisions to upgrade or downgrade the quality of the evidence using footnotes and added comments accordingly.

Judgements about the quality of the evidence were made by three review authors working independently, with disagreements resolved by discussion. Judgements were justified, documented and incorporated into the reporting of results for each outcome. ‘Summary of findings' tables were prepared for the primary endpoint.

When performing meta-analyses, a random-effects model was utilized to account for study heterogeneity. Forest plots were used to visualise the meta-analysis results.

Measures of treatment effect: Mean differences and standardised mean differences for continuous outcomes. All pain scales in the included studies were ranked 1 to 5, and 0 to 10. Results in scales of 1–5, were converted to 0 to 10 to allow pooling and more intuitive interpretation of results.

Dealing with missing data: When possible, we contacted the original investigators to request missing data. Contact was attempted twice using all retrieved e-mail address contacts.

Assessment of heterogeneity: We inspected forest plots visually to consider the direction and magnitude of effects and the degree of overlap between confidence intervals. We used the I^2^ statistics to measure heterogeneity among the trials in each analysis.

Assessment of reporting biases: We planned to create a funnel-plot if ≥ 10 studies were included. However, as the number of included studies was below this cut-off, this was not done.

We planned sub-analysis for treatment indication, number of study centres and study design (cross-over, other designs) when data for a specific local anaesthetic was available for ≥2 RCTs. Sensitivity analyses were planned for quality of evidence (RCTs with high-risk RoB domains vs. no high-risk domains).

### Role of the funding source

The funder supplied the research questions, defined the PICO (Population, Index test, Comparators, Outcomes) and had no role in study design, collection, analysis, and interpretation of data, or writing of the report. The funder commissioned independent reviewers who commented on the review's protocol and final report several times.

All authors had full access to the data in the study and had final responsibility for the decision to submit this manuscript for publication.

## Results

**Fig. 1:**
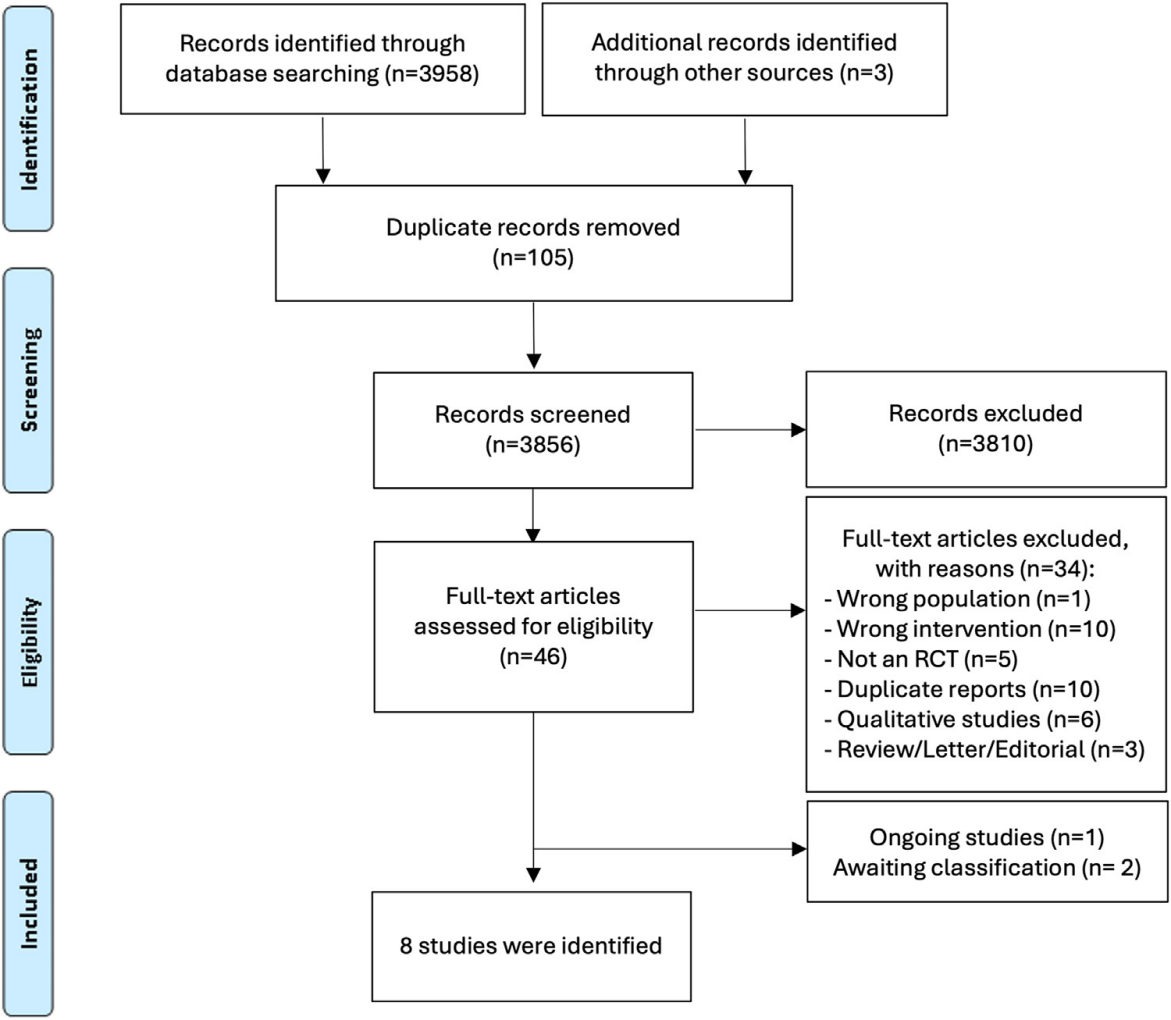
PRISMA flowchart. Legend: RCT—randomised controlled trial.

Database searches found a total of 3958 records, and 3 additional records were identified from grey literature searches. After removal of duplicates, and 3856 records were screened, and 46 reports were identified for full text review. Thirty-four were subsequently excluded due to: not being RCTs (n = 5), wrong population (patients given crystalline penicillin instead of BPG; n = 1), wrong intervention (e.g., cold/ice or manual pressure; n = 10), being duplicated reports (n = 10), qualitative studies (n = 6) or review/letters/editorials (n = 3) (Supplementary Table S1). The PRISMA flowchart is illustrated in Fig. 1. One ongoing study of potential interest was identified,^23^ and two additional studies^24^^,^^25^ were left as “awaiting classification” as they could not be excluded based on abstract, and the full-text was not available.

In the end, eight studies were included combining a total of 489 unique patients.^10^^,^^12^^,^^26^, ^27^, ^28^, ^29^, ^30^, ^31^ Bycroft et al.^26^ utilised Bicillin® LA and CR prefilled syringes. Farhadi and colleagues^27^ did not mention what type of intramuscular BPG was given. All remaining studies mixed the BPG powder with sterile water or lidocaine prior to the injection. Four studies utilized lidocaine 1% (3.2–4 mL) administered mixed in the intramuscular injection,^10^^,^^12^^,^^30^^,^^31^ one utilized lidocaine cream,^28^ one utilized 0.5 mL of 1% mepivacaine mixed in the intramuscular injection,^27^ and two utilized procaine penicillin G (0.6–1.2 M units of procaine penicillin G) mixed in the intramuscular injection^26^^,^^29^ (Table 1).

**Table 1:** Study design, population demographics, description of interventions and outcome assessment.

Study	Country	Design	Setting	Period	Participants/ Indication	Age	Female sex	Administered intervention	BPG dose	needle	Frequency of treatment	Pain assessment	Follow-up duration
Amir 1998	Israel	RCT with cross-over at 1 month Single-centre	Pediatric Ambulatory unit: Petah Tikva	November 1995 to March 1996	18 children 2ary prevention of ARF/RHD	Mean 14.9 (range: 11–19)	44% (8)	BPG diluted in 3.2 mL of sterile water vs. BPG diluted in 3.2 mL of 1% lidocaine hydrochloride	1.2 M Units	NA	Monthly	Visual “smile” pain scale from 1 to 5: - time of injection - at 2–4 h - at 24 h	2 months
Bycroft 2000	USA	RCT Two injections on the same day (one in each buttock) Single-centre	University children's and women's tertiary care ED: South Alabama	NA	50 pts (student body and house staff) GABS pharyngitis	Mean 32.0 (≥21 years)	NA	BPG (Bicillin® LA) 2 mL vs. penicillin G BPG + procaine mixture 2 mL (Bicillin® CR 0.6 M/0.6 M)	0.6 M–1.2 M Units adjusted to weight	21G	2 injections separated by one week	Visual analogue scale from 0 to 10: - immediately after - at 1 h - at 12 h	1 week
Estrada 2019	Spain	RCT—no crossover Multicentre	Two hospital sites/same city: Madrid	First half 2015	108 adults 1ary syphilis (41% with HIV): 55 pts mepivacaine vs. 53 controls	Mean: 36.6 ± 11 (≥18 years)	5.6% (14)	BPG diluted in 6 mL of sterile water vs. BPG diluted in 5.5 mL of sterile water + 0.5 mL of 1% Mepivacaine	2.4 M Units	19G (n = 52) 21G (n = 56)	Once only	Visual scale from 0 to 10: - immediately after - at 6 h - at 24 h	24 h
Farhadi 2010	Iran	RCT—no crossover Single-Centre	Outpatient clinic: Shirvan	NA	60 adults and young adults prescribed with BPG 30 pts lidocaine vs. 30 controls	35 Range 17–47	50% (30)	Lidocaine gel 2% applied 10 min before BPG injection	1.2 M Units	NA	Once only	Visual analogue scale from 0 to 10: - immediately after	immediate
Harari 1988	Papua New Guinea	RCT Two injections on the same day (one in each buttock) Single-centre	Adult casualty department of Goroka Base Hospital	1987	80 adolescents and young adults Moderate pneumonia	Estimated 20	NA	Procaine penicillin 1.2 M Units in 2 or 4 mL of sterile water and BPG 1 M Units in 2.3 mL (both diluted in sterile water) in each buttock	1.2 M Units Procaine Penicillin vs. 1.0 M Units BPG	23G	Once only	Asked which side was more painful: - immediately after - at 24 h	24 h
Jiamton 2022	Thailand	RCT Two injections on the same day (one in each buttock) Single-centre	Outpatient clinic: Bangkok	September 2018 to July 2019	40 adults Syphilis: 55% latent & 40% secondary stage; HIV in 57.7%	Mean 30.6 ± 10.3 (range: 18 to 59)	0% (0)	BPG diluted with 4 mL of lidocaine 1% or 4 mL of sterile water; changing buttock in different administration	1.2 M Units	20G	Three doses (assessment after first dose)	Visual analogue scale from 0 to 10: - during injection - immediately - at 5 min - at 20 min - at 24 h	24 h
Morsy 2012	Egypt & Saudi Arabia	RCT with cross-over at 1 month Multicentre	Two outpatient cardiology clinics: Sohag & Taibah	March to April 2011	100 children 2ary prevention of ARF/RHD	Mean 14.3 ± 2.4 (range: 10–19)	62% (62)	BPG diluted in 3.2 mL of sterile water vs. BPG diluted in 3.2 mL of 1% lidocaine hydrochloride	1.2 M Units	NA	Monthly	Visual pain scales using face chart - 0 to 10 (revised Faces pain scale) - immediate - after 2 h - at 24 h	2 months
Tamondong 2018	Philippines	RCT with cross-over at 21 days Single-centre	Outpatient clinic: Quezon City	2015	33 children 2ary prevention of ARF/RHD	14.3 ± 5.6 (Range: 10–18)	66.7% (22)	BPG diluted in 4 mL of sterile water vs. BPG diluted in 4 mL 1% lidocaine hydrochloride	0.6 M Units for <27.2 Kg 1.2 M Units for >27 Kg	21G	Every 3 weeks	Universal Pain Assessment Tool (UPAT), with facial grimace scale—0 to 10	3 weeks

NA—not available; RCT—randomised controlled trial; ARF—acute rheumatic fever; RHD—Rheumatic Heart Disease; BPG - benzathine penicillin G; NA—not available; ED—Emergency Department; GABS - group A beta-haemolytic streptococcal; HIV - human immunodeficiency virus; M− million; G—gauge needle; BPG - benzathine penicillin G.

Frequency and duration of intramuscular BPG treatment assessed in the different trials varied between single dose^27^, ^28^, ^29^ to three doses,^30^ and interval ranged from weekly^26^ to three^31^ to four-weekly^10^^,^^12^ (Table 2).

**Table 2:** Summary of Observed and pooled results for pain with different interventions.

Intramuscular Lidocaine vs. Placebo					
Study	Timing	Intervention	Control	Mean Difference	P value/ I2
Amir 1998	Immediately after	3.4 ± 2.5	7.4 ± 2.5	−4.00 [−5.63 to −2.37]	0.0002
Jiamton 2022	Immediately after	2.25 ± 1.85	7.93 ± 1.95	−5.68 [−6.51 to −4.85]	<0.0001
Morsy 2012	Immediately after	5.2 ± 0.7	6.7 ± 1.0	−1.50 [−1.74 to −1.26]	<0.0001
Tamondong 2018	Immediately after	0.63 ± 1.03	4.88 ± 2.23	−4.25 [−5.09 to −3.41]	<0.0001
Pooled	Immediately after			−3.84 [−6.19 to −1.48]	P = 0.001 I2 = 98%
Jiamton 2022	5 min	0.60 ± 1.43	3.45 ± 2.64	−2.85 [−3.78 to −1.92]	<0.0001
Jiamton 2022	20 min	0.35 ± 1.21	2.20 ± 2.15	−1.85 [−2.61 to −1.09 ]	<0.0001
Amir 1998	2–4 h	3.8 ± 2.0	4.8 ± 2.0	−1.00 [−2.63 to 0.63]	0.23
Morsy 2012	After 2 h	3.8 ± 1.3	4.0 ± 1.3	−0.20 [−0.67 to 0.27]	0.41
Pooled	After 2 h/ 2–4 h			−0.26 [−0.71 to 0.19]	P = 0.26 I2 = 0%
Amir 1998	24 h	3.5 ± 0.7	3.3 ± 0.5	0.20 [−0.20 to 0.60]	0.32
Jiamton 2022	24 h	2.80 ± 1.94	3.13 ± 2.08	−0.33 [−1.21 to 0.55]	0.16
Morsy 2022	24 h	1.2 ± 0.3	1.2 ± 0.3	0 [−0.08 to 0.08]	1.00
Pooled	24 h			0.01 [−0.08 to 0.09]	P = 0.89 I2 = 0%

Lidocaine skin cream vs. No intervention					
Study	Timing	Intervention	Control	Mean difference	P value
Farhadi 2010	Immediately after	6.85 ± 1.05	7.39 ± 1.55	−0.54 [−1.17 to 0.09]	0.13

Intramuscular Procaine vs. Placebo					
Study	Timing	Intervention	Control	Mean difference	P value
Bycroft 2000	Immediately after	Lower^a^	a	–	0.0001
Bycroft 2000	1 h	Lower^a^	a	–	0.008
Bycroft 2000	12 h	No differences^a^	a	–	0.76

Intramuscular Mepivacaine vs. Placebo					
Study	Timing	Intervention	Control	Mean difference	P value
Estrada 2019	Immediately after	3.15 ± 1.10	5.34 ± 1.15	−2.19 [−2.49 to −1.89]	<0.0001
Estrada 2019	6 h	2.21 ± 0.90	2.30 ± 1.00	−0.09 [−0.34 to 0.16]	0.49
Estrada 2019	24 h	1.81 ± 1.35	1.84 ± 1.25	−0.03 [−0.38 to 0.32]	0.87

a Values for each of the 3 time periods were not provided in the publication.

The studies were conducted over more than four decades (published between 1988 and 2022) and in multiple continents and geographic areas (Table 1). Three studies included children and/or young adults on secondary prevention for RHD,^10^^,^^12^^,^^31^ one study treated adults with group A Beta-haemolytic streptococci pharyngitis,^26^ one study treated adolescents with moderate pneumonia,^29^ one included adults and provided no indication for BPG treatment,^28^ and the remaining two included adults with Syphilis.^27^^,^^30^

One study included two centres,^27^ and all other studies were single-centre. Three studies were RCTs with cross-over at 21 days^31^ or 1 month,^10^^,^^12^ and three studies involved two injections on the same day (one in each buttock).^26^^,^^29^^,^^30^

BPG dose was 1.2 M Units for most studies, except for Harari and colleagues,^29^ where 1.0 M Units were used, and Estrada and colleagues^27^ which used 2.4 M Units. Two studies^26^^,^^31^ adjusted the dose to 0.6 M Units for individuals with lower body weight (Table 1). Follow-up ranged from 24 h^27^^,^^29^^,^^30^ or less,^28^ to a few weeks^26^^,^^31^ or two months.^10^^,^^12^ Six studies utilized pain scales ranging from 1 to 10^10^^,^^26^, ^27^, ^28^^,^^30^^,^^31^ or 1 to 5.^12^ In one study, participants were only asked which buttock less painful.^29^

**Fig. 2:**
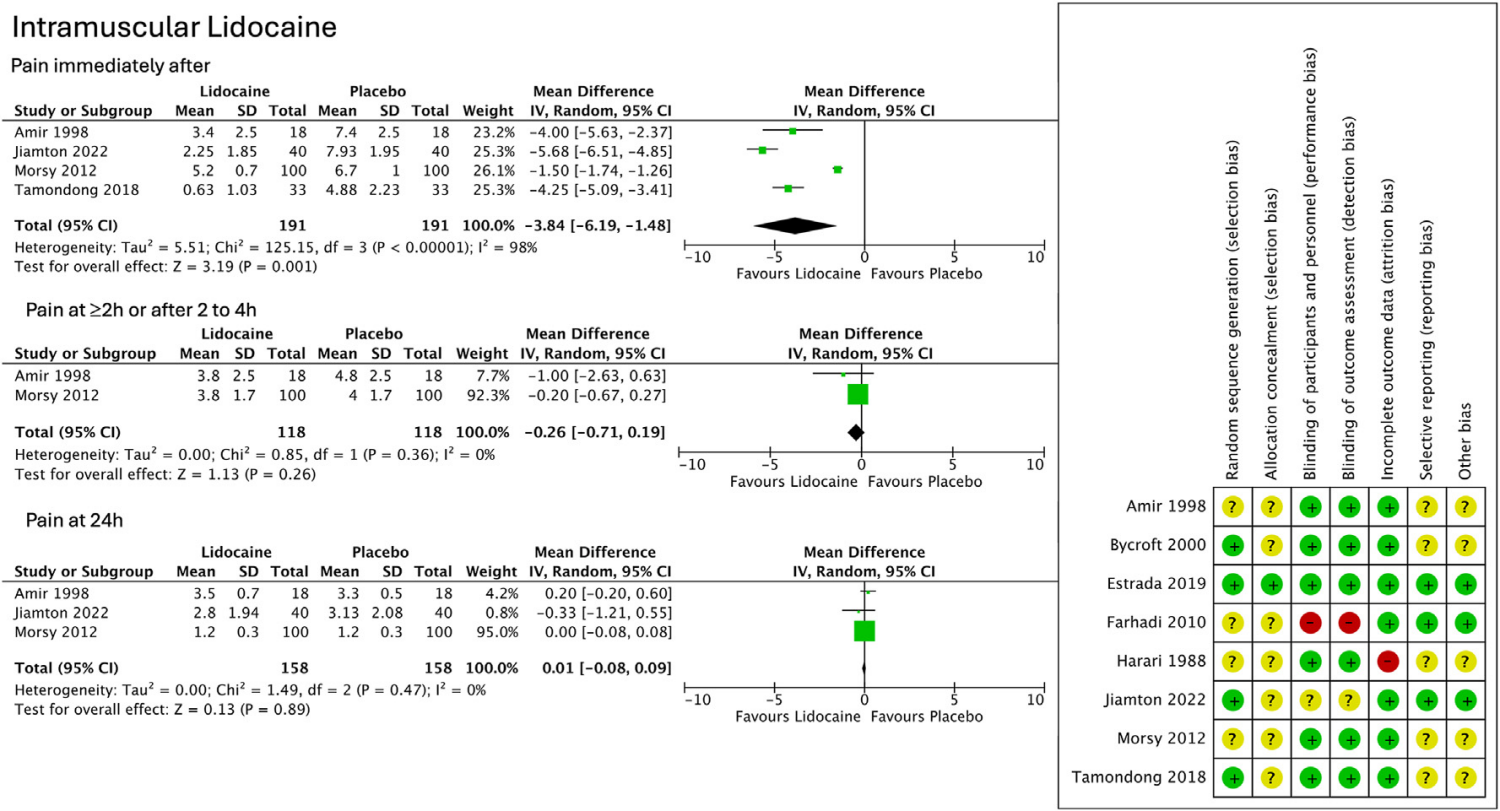
Left Panel. Forest-plots with pooling of data on intramuscular lidocaine; Right Panel—Risk of Bias assessment of included trials. Legend: SD—standard deviation; CI—confidence interval; IV—inverse variance.

Only one study had domains with high-risk of bias (lack of blinding),^28^ and one study was considered low risk of bias for all domains.^27^ Three studies had information on trial protocol registration^27^^,^^28^^,^^30^ (Fig. 2). Frequent lack of information on the randomization process and lack of published trial protocols resulted in most studies being classified as unclear risk for such selection bias, reporting bias and other bias. A detailed assessment of Risk of Bias and judgements is provided in Supplementary Table S2.

Pain immediately after the injection was more intense, with the mean reported value in the control group ranging from 4.88 to 7.93 on a scale of 0–10. Mean pain levels dropped to approximately half of these levels in the next 5 min to first 2–6 h and remained in the range of 1.2–3.3 at 24 h (Table 2).

Pain levels were significantly lower in patients treated with lidocaine mixed with BPG and administered with intramuscular injection in studies assessing pain immediately after the injection,^10^^,^^12^^,^^30^^,^^31^ and at 5–20 min.^30^ Amir and colleagues reported that on completion of the RCT, 70.6% of children reported feeling less pain with lidocaine, and the remaining 29.4% denied feeling any difference between the 2 injections.^12^ Pooled data for the 4 studies assessing the effect of lidocaine immediately after the intramuscular injection showed a significant reduction (mean difference of −3.84, 95% CI −6.19 to −1.48, P = 0.001) when compared to controls (Fig. 2 & Table 2). Heterogeneity was high (I^2^ = 98%) due to the broad variation in point estimates and 95% CI. No significant differences were observed for the subsequent time points of this comparison (Pooled results for 2 h to 2–4 h: mean difference = −0.26, 95% CI −0.71 to 1.9, P = 0.26; I^2^ = 0%; pooled results at 24 h: mean difference = 0.01, 95% CI −0.08 to 0.09, P = 0.89; I^2^ = 0%).

One study^28^ failed to demonstrate a benefit of lidocaine cream on the reduction of immediate pain.

Bycroft et al. assessed intramuscular BPG mixed with procaine and reported a significant reduction in immediate and 1 h pain levels,^26^ failing to provide the effect estimate for the different assessed timepoints. When comparing the pooled scores for the three measured timepoints within the same individuals (i.e., comparison of buttock injected with procaine BPG vs. buttock injected with BPG), mean pain scores for the buttock treated with procaine BPG were significantly lower (P < 0.001).

Harari and colleagues also assessed the impact of mixing procaine with BPG. All patients receiving 1.2 M Units of Procaine BPG in a 4 mL dilution reported that this buttock was less painful than the one treated with 1.0 M Units of BPG in 4.3 mL dilution (P < 0.001).^29^ Among patients treated 2.4 M Units of procaine BPG in a 4 mL dilution, the majority (78%) reported more pain in the buttock injected with BPG (P < 0.001). On that study, approximately half of patients were follow-up at 24 h, and 62%–69% still reported pain, with 100% to 82 of these (respectively the comparison vs. diluted and concentrated BPG procaine), locating pain to the buttock injected with BPG.

Estrada and colleagues reported a significant reduction in immediate pain among patients treated with mepivacaine mixed with BPG, with no significant benefit observed 6 h and 24 h post injection.^27^

**Fig. 3:**
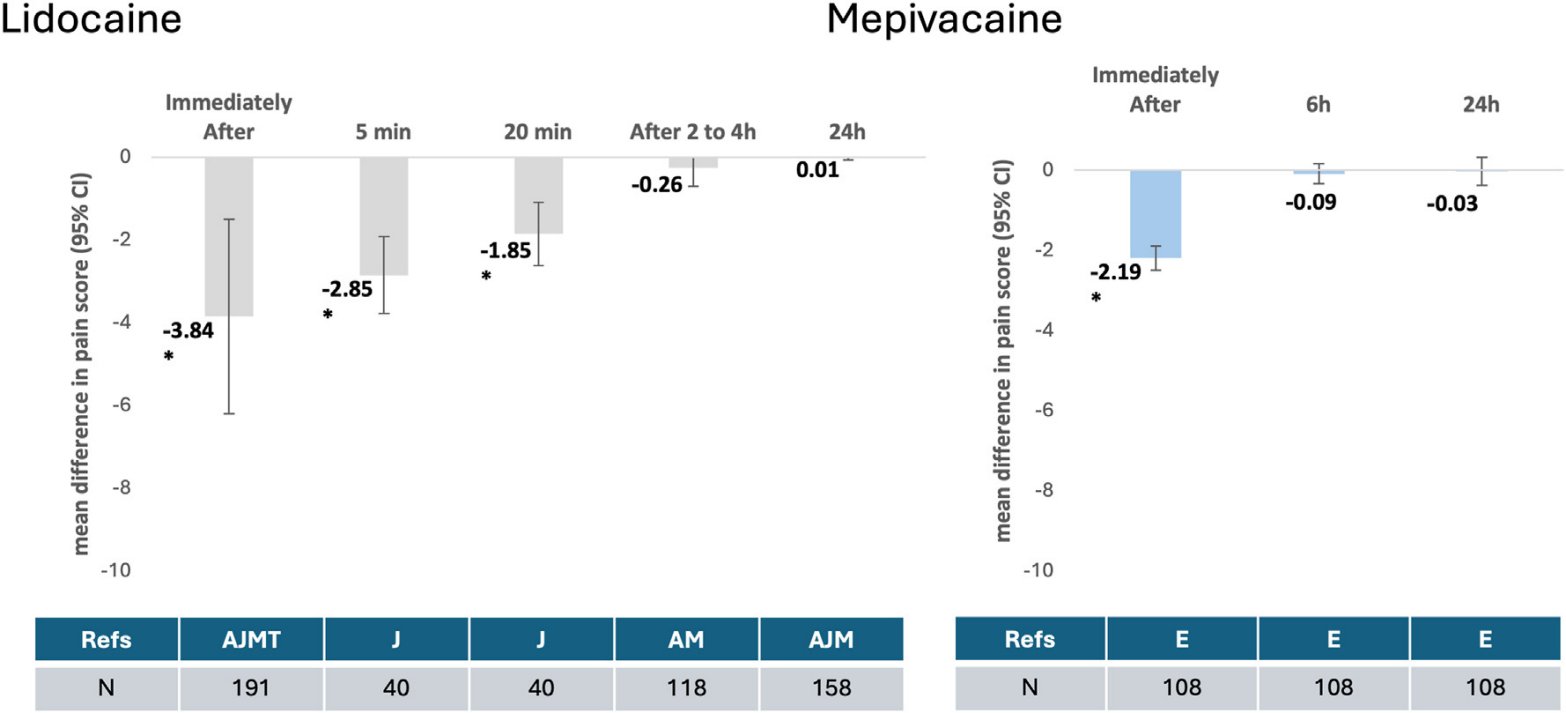
Time-dependent effect (mean difference with 95% CI confidence intervals) of Lidocaine (left) and Mepivacaine on pain post-intramuscular BPG injection. Legend: BPG - benzathine penicillin G; N—number of patients; A - Amir J et al. 1998^10^; J—Jiamton S et al., 2022^28^; M− Morsy MM et al. 2012^8^; T—Tamondong RM et al. 2018^29^; E—Estrada V et al. 2019.^25^

Fig. 3 illustrates the variation of effect of lidocaine and mepivacaine compared to placebo assessed at the different timepoints.

Sub-analyses of intramuscular lidocaine RCTs^10^^,^^12^^,^^30^^,^^31^ were performed for indication for BPG, number of centres and study design, but results were not different from the main analysis (Supplementary Table S3). No sensitivity analyses were performed for quality of evidence, as no high-risk domains in RoB were present for intramuscular lidocaine trials.

Quality of evidence assessed through the GRADE framework was considered moderate for reduction of immediate pain, and pain assessed at 5 and 20 min when injecting lidocaine mixed with BPG (Table 3). On the other hand, evidence for lack of pain reduction benefit with intramuscular lidocaine at 2 h and after was considered high quality. Quality of evidence for lidocaine cream as classified as low, for intramuscular mepivacaine was classified as moderate, and for procaine it was considered as low quality.

**Table 3:** Summary of findings with GRADE.

Outcome	Effect size intervention vs control 95% CI P	Studies sample size	Heterogeneity	Risk of bias assessment	Imprecision	Indirectness publication bias	Interpretation quality of evidence/GRADE
Intramuscular lidocaine							
Pain immediately after	MD= −3.84 [−6.19 to −1.48] P = 0.0001	4 RCTs 191 pts	High heterogeneity 98% (↓1 level)	Low risk No high-risk domains	Broad 95% CI but no downgrade^a^	No indirectness –b	Significant reduction ⊕⊕⊕◯ Moderate-quality
Pain at 5 min	MD = −2.85 [−3.78 to −1.92] <0.0001	1 RCT 40 pts	–c	Low risk No high-risk domains	No imprecision	Indirectness (↓1 level)^d^ –	Significant reduction ⊕⊕⊕◯ Moderate-quality
Pain 20 min	MD = −1.85 [−2.61 to −1.09] <0.0001	1 RCT 40 pts	–	Low risk No high-risk domains	No imprecision	Indirectness (↓1 level) –	Significant reduction ⊕⊕⊕◯ Moderate-quality
Pain after 2 h/ 2 to 4 h	MD = −0.26 [−0.71 to 0.19] P = 0.26	2 RCTs 118 pts	No heterogeneity 0%	Low risk No high-risk domains	No imprecision	No indirectness –	No reduction ⊕⊕⊕⊕ High quality
Pain at 24 h	MD = 0.01 [−0.08 to 0.09] P = 0.89	3 RCTs 158 pts	No heterogeneity 0%	Low risk No high-risk domains	No imprecision	No indirectness –	No reduction ⊕⊕⊕⊕ High quality
Lidocaine skin cream							
Pain immediately after	MD= −0.54 [−1.17 to 0.09] P = 0.13	1 RCT 60 pts	–	Two high-risk domains (↓1 level)	Imprecision^e^ (↓1 level)	No indirectness –	No reduction ⊕⊕◯◯ Low quality
Intramuscular mepivacaine							
Pain immediately after	MD= −2.19 [−2.49 to −1.89] P < 0.0001	1 RCT 108 pts	–	Low risk No high-risk domains	No imprecision	Indirectness (↓1 level) –	Significant reduction ⊕⊕⊕◯ Moderate-quality
Pain at 6 h	−0.09 [−0.34 to 0.16] P = 0.49	1 RCT 108 pts	–	Low risk No high-risk domains	No imprecision	Indirectness (↓1 level) –	No reduction ⊕⊕⊕◯ Moderate-quality
Pain at 24 h	−0.03 [−0.38 to 0.32] P = 0.87	1 RCT 108 pts	–	Low risk No high-risk domains	No imprecision	Indirectness (↓1 level) –	No reduction ⊕⊕⊕◯ Moderate-quality
Intramuscular procaine							
Pain immediately after	Less pain NA^e^ P = 0.001	1 RCT 80 pts	–	Low risk No high-risk domains	No data on 95% CI (↓2 levels)	No indirectness –	Significant reduction ⊕◯◯◯ Low-quality
Pain at 1 h	Less pain NA P = 0.008	1 RCT 80 pts	–	Low risk No high-risk domains	No data on 95% CI (↓2 levels)	No indirectness –	Significant reduction ⊕◯◯◯ Low-quality
Pain at 12 h	No differences P = 0.76	1 RCT 80 pts	–	Low risk No high-risk domains	No data on 95% CI (↓2 levels)	No indirectness –	No reduction ⊕◯◯◯ Low-quality

MD—mean difference; CI—confidence interval; RCT—randomized controlled trials.

a Decision not to downgrade as this was already done for high heterogeneity, which is contributing to the broad 95% CI in the magnitude of pain reduction.

b Publication bias—not assessed as <10 RCTs per outcome.

c Heterogeneity not available for outcomes with data from only 1 RCT.

d Only patients with Syphilis receiving BPG.

e No data available on effect size and 95% CI for each of the three time periods - decision to downgrade by two levels as unable to assess the magnitude of imprecision.

No studies assessed the following endpoints: fear, adherence, or acceptability to provider and patient.

Two studies reported absence of any relevant adverse reactions.^12^^,^^31^ Estrada et al.^27^ and Harari et al.,^29^ upon e-mail contact, confirmed that no adverse events were observed in their studies. Jiamton reported adverse events in 15 patients (37.5%): 9 had generalized rash, 8 generalized pruritus and 8 had fever, and one patient experienced a “minor drug allergy”, but the authors did not clarify with each treatment these were observed.^30^ Bycroft and colleagues described a 20-min observation period for potential adverse reactions after administration of BPG but failed to report any events.^26^ The two remaining studies^10^^,^^28^ did not assess, or failed to report, any adverse events.

## Discussion

Our findings suggest that mixing local anaesthetics (lidocaine, mepivacaine or procaine) with BPG may significantly improve the reported immediate pain level, or pain experienced during the first minutes following intramuscular BPG. The observed reduction in pain levels meets accepted criteria for a moderate to major clinical meaningful difference (i.e., a reduction of 2 points on a 10-point pain scale, or a 30% reduction vs. comparator, is considered a moderately important difference; a reduction of 4 points, or a 50% reduction is considered a major improvement).^32^^,^^33^ Quality of evidence is higher for lidocaine and mepivacaine, than for procaine. No severe adverse reactions were reported. These findings are of interest to patients treated with BPG, such as those in the included trials (patients on secondary prevention for ARF/RHD, or receiving treatment for syphilis or Streptococcal infections), for whom injection-related pain will be an issue. Intensity of pain associated with intramuscular BPG administration is an important consideration for secondary prevention RHD programmes, or indications where multiple BPG injections are required (e.g., syphilis). Immediate pain level, as reported by patients, was of high intensity in most studies. Low intensity pain was still reported at 24 h.

Despite being acutely effective, local anaesthetics were not effective and reducing pain hours after the injection. This finding is in agreement with the duration of action of the assessed local anaesthetics (lidocaine: half an hour to 3 h; mepivacaine: 1.5–2 h; and procaine: 45–60 min). Similarly, lidocaine cream failed to reduce immediate pain level in the only study assessing this local anaesthetic formulation.^28^ The effect of topical lidocaine depends on contact time: 60 min are associated with numbness to a depth of 3 mm, and 120 min associate with numbness to a depth of 5 mm.^34^ It is therefore possible that, either more time is required for lidocaine cream to exert its action in the setting of intramuscular BPG injection, or that, possibly, lidocaine cream may not be suited for this particular clinical scenario (i.e., the site of intramuscular injection is deeper than the depth of action that can be achieved with topical lidocaine).

Besides local anaesthetics, other alternatives have been identified in the search for reducing injection pain: Buzzy®, Shotblocker®, virtual reality and distraction cards, manual pressure or cold before injection, and alternative locations for injection (Supplementary Table S1). The Shotblocker® is a plastic C-shaped device with small bumps on is back which, when pressed against the skin, saturates the sensory signals around the injection site and distracts patients from the pain signals of the needle poke. The Buzzy® is a vibrating bee-shaped gadget with blue icepack wings which reduces needle-related pain through the combination of vibration and cold. The Australian RHD guidelines suggest a stepwise approach with strategies for managing injection pain, fear and distress^35^: direct pressure, cold pack, cold needle, vibration device or oral paracetamol are recommended for most patients; local anaesthesia with lidocaine is recommended for patients with pain issues, and clonidine or nitrous oxide are recommended for patients with phobias or uncontrolled pain.^35^

No quantitative evidence was found regarding a potential effect of local anaesthetics on increasing treatment adherence or reducing fear or anxiety levels, and no studies assessed provider or patient preference. A qualitative study of 29 aboriginal children and 59 clinicians in Australia has looked into this matter, and suggested that not only are injections perceived as very painful by many patients (frequently described as “difficult to bear”), but also BPG injections cause distress and sorrow among healthcare professionals.^36^ This is of relevance as it may cause healthcare professionals to feel reluctant to administer monthly BPG injections.^36^ Another important aspect raised by Mitchell and colleagues was the insufficient information of patients regarding their medication management and the possibility use of pain reduction measures. Even though some patients showed ability to negotiate this aspect with clinicians, and accepted an offer of local anaesthetic, other patients demonstrated either lack of ability or resignation with pain. Furthermore, some patients reported they were not consistently offered pain relief.^36^ Data from focus groups comprising 36 patients on monthly BPG injections suggest that barriers to adherence to secondary prevention include fear of injection pain, poor patient-provider communication, poor availability of clinics and providers able to give injections, and lack of resources for transportation and medications. The key facilitators identified in the study were: perception of worsening of disease with missing injections, positive relationship with health care providers, a reminder system for injections, personal motivation and supportive family and friends.^37^ The painful nature of BPG injections was also identified as an important reason for non-adherence in patients with RHD from Uganda.^38^

No data was available on the costs and feasibility of utilizing local anaesthetics in secondary prevention of RHD programmes. Additional costs due to local anaesthetics and the short preparation time may need to be factored.

Utilized doses of lidocaine (30–40 mg) and mepivacaine (5 mg) were far below the known toxicity limit (3–4.5 mg/kg). For procaine, higher doses were used as the maximum recommended dose for procaine penicillin G in individuals weighting >27 Kg is 2.4 M Units, 13.5 to 27 Kg is 1.2 M Units and <13.5 Kg is 0.6 M Units). However, attention to specific patient factors (e.g., presence of liver disease), besides weight is required when using these agents. Lidocaine 1% (10 mg/mL) considered to have equivalent efficacy to mepivacaine 1%–1.5% (10–15 mg/mL). Procaine is less potent, with procaine 2% (20 mg/mL) being considered equivalent to lidocaine 1%.^39^

Pain scales were utilized to measure pain levels. These are subjective and may vary from person to person. Furthermore, as pain scales focus on perceived pain at the moment of the test, they may be influenced contextual factors (e.g., the current state of mind and life events on that day). Finally, pain scales may fail to capture important aspects such as pain fluctuations over time, how activity changes pain, threshold of pain tolerance, pain history, cultural differences, and emotional state.^40^

Some additional limitations need to be highlighted in this systematic review. Most studies included small patient samples, and a nearly half of studies failed to report adverse events. Some RCTs had a cross-over design, but as they were all double-blind, and patients were already on chronic monthly BPG injections, we do not believe this to have been a source of bias. Furthermore, sensitivity analysis for studies with and without cross-over showed comparable results (Supplementary Table S3). The included studies did not allow us to assess the impact of local anaesthetics’ dose on pain intensity and duration of pain relief. Further trials are required to address this matter. Furthermore, different injection volumes were used across studies (e.g., 2 mL, 2.3 mL, 4.2 mL and 6 mL). Injection volume may impact on pain, and it is possible it may explain some of the observed heterogeneity. Unfortunately, the number of included studies was small (less than 10), and we were not able to perform a meta-regression. Finally, less than half of the patients in this systematic review had RHD. However, we did not observe any differences on the effect of local anaesthetics when analysing our data by treatment indication. This is particularly important as patients who are treated with a single intramuscular BPG injection (i.e., patients treated for early syphilis) may experience pain differently than patients who have been on monthly BPG for years (patients with ARF and RHD). Being on regular BPG may affect pain perception and it is possible that, in some cases, it can cause anxiety and further aggravate the pain.

More research is warranted on the impact of lidocaine in anxiety and fear of BPG injection, as well as treatment adherence. Health professionals and patient preference should be assessed in more detail in future trials. Data on the safety of this approach are scarce, and study authors should be encouraged to report side effects, or the absence of side effects.

Other pain reduction strategies such as watching three-dimensional videos with virtual reality glasses,^41^ manual pressure before the injection,^42^ local cold/ice,^43^ or the Buzzy®^44^ have been associated with clinically meaningful reduction in pain intensity. Unfortunately, no randomized controlled trials have compared these strategies with local anaesthetics. Further research into this area would be of interest to address whether local anaesthetics have a clear advantage over these options and/or if there is a role for combination of different pain management strategies for optimizing clinical benefit (e.g., local anaesthetics and cold, or local anaesthetics and Buzzy).

Further trials are required to clarify combination of pain management options. Russel and colleagues have previously suggested, in a non-randomised study, that Buzzy combined with intramuscular lidocaine may lead to a more pronounced reduction in pain levels than the use of lidocaine in isolation.^18^ A different area of research is the use of subcutaneous BPG, with or without lidocaine.^45^^,^^46^ Preliminary data suggests that subcutaneous BPG administration can potentially allow spacing of BPG administration to once every three months.^47^

In conclusion, in patients receiving intramuscular BPG injections, moderate quality quantitative evidence suggests that BPG injections diluted with lidocaine or mepivacaine may improve post-injection pain scores compared to BPG injections diluted with sterile water. Procaine may also have a benefit, but quality of evidence was lower. Despite the scarcity of data, use of local anaesthetics in the setting of secondary RHD prevention appears to be safe.

## Contributors

FP and RP wrote the final draft of the manuscript. FP & RP provided methods input, and alongside with AA, BK, NA, SA & YK accessed the raw data and verified it. FS and FP provided information specialist expertise. MA, JJHB, EM, MC, and DC provided clinical input. All authors revised the first draft of the manuscript and provided comments to improve it and prepare the final version. All authors read and approved the final version of the manuscript and agreed with submission for publication.

## Data sharing statement

All utilized data for the analyses was extracted from the included studies and are included in the Article or uploaded as Supplementary information. No patient-level data was utilized.

## Declaration of interests

All authors declare no competing interests.

## Appendix ASupplementary data

Appendix 1 and 2 and Tables S1–S3

Abstract Persian

Abstract Urdu

Abstract French

Abstract in Spanish

Abstract portugues
