# Supplementary material for: Local anaesthetic to reduce injection pain in patients who are prescribed intramuscular benzathine penicillin G: a systematic review and meta-analysis
Source: eClinicalMedicine. 2024 Sep 4;76:102817. doi: 10.1016/j.eclinm.2024.102817 (PMC11404083; doi:10.1016/j.eclinm.2024.102817)
Supplement: Appendix 1 and 2 and Tables S1–S3 [file mmc1.pdf]

Supplementary Material

Ferruccio Pelone, Bessie Know, Sabahat Ahmed, Yakup Kilic, Ahsan Ali, Nida Ahmed, Mahmood Ahmad, Jonathan JH Bray, Farhad Shokraneh, Miryan Cassandra, David S Celermajer, Eloi Marijon, Rui Providencia. Local anaesthetic to reduce injection pain in patients who are prescribed intramuscular benzathine penicillin G: a systematic review. eClinicalMedicine. 2024

Index

Appendix 1: Benzathine penicillin G dosing regimens and indications……………………………..2

BPG formulations………………………………………………………………………………………3

Appendix 2: Search strategy……………………………………………………………………………………..4

Table S-1: Table of excluded studies…………………………………………………………………………..5

Table S-2. Risk of Bias assessment………………………………………………………………………………..8

Table S-3. Sub-analyses and Sensitivity analyses…………………………………………………………….11

Appendix 1: Benzathine penicillin G (BPG) dosing regimens and indications

	WHO	AHA
Prophylaxis against recurrent rheumatic fever	<30 kg: 0.6MU IM<br>>30 kg: 1.2MU IM<br><br>Every 3 to 4 weeks (shorter spacing for high risk)<br><br>Duration<br>-No evidence of carditis: 5 years since last attack or 18 years old (whichever is longest)<br>-Resolved carditis: 10 years since last attack or 25 years old<br>-Moderate-severe RHD or surgery: lifelong	≤27 kg: 0.6MU IM<br>>27 kg: 1.2MU IM<br><br>Every 4 weeks (3-week regimens can be considered for high risk or patients with recurrent ARF despite high-adherence to 4-weekly schedule)<br><br>Duration<br>- No evidence of carditis: 5 years or until 21 years of age (whichever is longest)<br>- Carditis without residual disease: 10 years or until 21 years of age (whichever is longest)<br>- Carditis with residual heart disease: 10 years or until 40 years of age (whichever is longer), sometimes lifelong;
	RHDAustralia	AHA
Treatment of Streptococcal tonsillopharyngitis in if high-risk for RF and potential issues with adherence to therapy	<10 Kg: 0.45 MU IM<br>10- to<20 kg: 0.6MU IM<br>≥20 kg: 1.2MU IM<br>Single dose	≤27 kg: 0.6MU IM<br>>27 kg: 1.2MU IM<br>Single dose
	RHDAustralia	-
Streptococcal Impetigo and Cellulitis	<10 Kg: 0.45MU IM<br>10- to<20 kg: 0.6MU IM<br>≥20 kg: 1.2MU IM<br>Single dose	-
	WHO	CDC
Early syphilis<br>- Primary syphilis<br>- Secondary syphilis<br>- Early latent syphilis	BPG 2.4MU IM* in a single dose;<br>Children**	BPG 2.4 MU IM in a single dose**;
Late syphilis<br>- Tertiary syphilis with normal CSF ***<br>- Late latent syphilis	BPG 7.2 MU total, administered as 3 doses of 2.4 MU IM each at 1-week intervals;	BPG 7.2 MU total, administered as 3 doses of 2.4 MU IM each at 1-week intervals;

Legend: WHO – World Health Organization; CDC - Centers for Disease Control and Prevention; MU- million units; IM – intramuscular; AHA – American Heart Association; CSF – cerebrospinal fluid; * The 2.4MU IM dose is usually divided into two injections of 1.2MU each administered into each of the gluteal muscles; ** Syphillis' BPG dose for children: 50,000 units/kg body weight IM, up to the adult dose of 2.4 MU, in a single dose; *** for adults with Neurosyphilis, Ocular syphilis or Otosyphilis, aqueous crystalline penicillin G 18–24 MU per day, administered as 3–4 MU IV every 4 hours or continuous infusion for 10–14 days is recommended.

BPG formulations

- Lyophilised powder packaged in vials, which needs to be mixed with sterile diluent at point of care.
- Viscous liquid in pre-filled syringes that need to be stored on a refrigerator (temperature 2 to 8ºC); Bicillin® L-A (Pfizer) is available in three dosages: 0.6MU (1.17mL), 1.2MU (2.3mL) and 2.4MU (4.6mL)

Note: During a recent period of shortage of Bicillin® L-A in Australia, guidance has been issued by Menzies school of health research, National Aboriginal Community Controlled Health Organization, and the Northern Territory Government, on the preparation of BPG 1.2MU powder for suspension:

- Reconstitute the powder into a suspension with either:
    - 4.0mL of Lidocaine 1%
        - or
    - 4.0mL of sterile water
- Carefully shake for at least 20 seconds until a smooth suspension is obtained
- Wait for 30s for any bubbling of the solution to subside
- Administer immediately after these steps
- A maximum volume of 5mL can be injected into a muscle in a single injection

References

Gerber MA, Baltimore RS, Eaton CB, Gewitz M, Rowley AH, Shulman ST, Taubert KA. Prevention of rheumatic fever and diagnosis and treatment of acute Streptococcal pharyngitis: a scientific statement from the American Heart Association Rheumatic Fever, Endocarditis, and Kawasaki Disease Committee of the Council on Cardiovascular Disease in the Young, the Interdisciplinary Council on Functional Genomics and Translational Biology, and the Interdisciplinary Council on Quality of Care and Outcomes Research: endorsed by the American Academy of Pediatrics. Circulation. 2009;119:1541-51. doi: 10.1161/CIRCULATIONAHA.109.191959.

NT Health, Menzies School of Health Research, and National Aboriginal Community Controlled Organisations. Preparation and Dosing of Long-acting S19A Benzathine Benzylpenicillin EXTENCILLINE® Product. May 2024. Available at: https://www.rhdaustralia.org.au/system/files/fileuploads/s19a_benzathine_benzylpenicillin_extencilline_preparation_and_dosing_information_resource_may_2024.pdf , last accessed 5th July 2024

RHDAustralia (ARF/RHD Writing Group). The 2020 Australian guideline for prevention, diagnosis and management of acute rheumatic fever and rheumatic heart disease. 3.2 edition, March 2022; (2020). Available at: https://www.rhdaustralia.org.au/system/files/fileuploads/arf_rhd_guidelines_3.2_edition_march_2022.pdf , last accessed 4th July 2024

Workowski KA, Bachmann LH, Chan OA, Johnston CM, Muzny CA, Park I, Reno H, Zenilman JM, Bolan GA. Sexually Transmitted Infections Treatment Guidelines, 2021. MMWR Recomm Rep 2021;70:19-60.

World Health Organization. Guidelines for the management of symptomatic sexually transmitted infections. Geneva: World Health Organization; 2021. Available at: https://www.who.int/publications/i/item/9789240024168 , last accessed 4th July 2024

World Health Organisation. Rheumatic Fever and Rheumatic Heart Disease: Report of a WHO Expert Consultation, Geneva, 29 October–1 November 2001; 2001. Available at apps.who.int/iris/bitstream/handle/10665/42898/WHO_TRS_923.pdf?sequence=1&isAllowed=y , last accessed 13th May 2024

Wyber R, on behalf of RHD Action. Global Status of BPG Report. RHD Action. 2016. https://rhdaction.org/resources/global-status-bpg-report , last accessed 4th July 2024

Appendix 2: Search strategy

Database: Ovid MEDLINE(R) ALL <1946 to May 2, 2024>

1. ("pain" and "penicillin").mp. [mp=title, book title, abstract, original title, name of substance word, subject heading word, floating sub-heading word, keyword heading word, organism supplementary concept word, protocol supplementary concept word, rare disease supplementary concept word, unique identifier, synonyms, population supplementary concept word, anatomy supplementary concept word] (999)

2. limit 1 to humans (831)

Database: EMBASE <1947 to May 2, 2024>

1. ("pain" and "penicillin " and "trial").mp. [mp=title, abstract, heading word, drug trade name, original title, device manufacturer, drug manufacturer, device trade name, keyword heading word, floating subheading word, candidate term word](8399)

2. limit 1 to (human and "remove medline records") (2945)

Conference Proceedings Citation Index-Science (CPCI-S; 1990 - to May 2, 2024)

1. "pain" and "penicillin" (20)

Cochrane Central Register of Controlled Trials (Issue 4 of 12, April 2024)

1. "pain" and "penicillin G" and "trial"" (320)

2. exclude EMBASE & Medline (117)

Latin American and Caribbean Health Sciences Literature (LILAC; 1990 - to 7th April 2024)

“Title, Abstract, Subject”

1. "pain" and "penicillin" (45)

Table S-1: Table of excluded studies

Study name	Reason for exclusion
Adnan et al. 2013	Not an RCT; controlled study assessing patients' experience following 3 monthly im BPG injections: first diluted in water 2nd and 3rd diluted in lidocaine.
Basak et al. 2021	Wrong intervention; RCT assessing impact of virtual reality and distraction cards on pain associated with im BPG injection.
Cooper et al. 2023	Qualitative study describing experiences of young RHD patients receiving subcutaneous lidocaine 2% followed by subcutaneous BPG.
Derya et al. 2015	Wrong intervention; RCT assessing impact of manual pressure before im BPG injection on pain.
Douglas et al. 1971	Wrong population: the control group was treated with crystalline penicillin 500,000U every 6h for 5 days (short duration of action form of Penicillin G); study not assessing pain.
Enkel et al. 2023	Qualitative study describing experiences of RHD receiving different subcutaneous doses of BPG
Farhadi et al. 2011	Wrong intervention; RCT assessing impact of cold/ice cubes on pain associated with im BPG injection.
Feamley et al. 2020	Not an RCT; Wrong intervention: comparison of single 8mL versus double injection of 4mL im BPG in patients with Syphilis (treatment arm chosen by patients).
Hla et al. 2024	Review paper on subcutaneous BPG for Syphilis.
Huck et al. 2015	Qualitative study; Assessment of factors influencing adherence to secondary prophylaxis.
Janier et al. 2012	Not an RCT; Wrong intervention: comparison of single versus multiple im BPG doses
Jakhwal et al. 2018	Wrong intervention; Ongoing RCT assessing impact of Helfer Skin Tap on pain associated with im BPG injection.
Kado et al. 2020	Wrong intervention; RCT comparing pharmacokinetic profile and tolerability of subcutaneous versus im BPG
Kado et al. 2023	Wrong intervention; RCT assessing 3 doses of subcutaneous BPG and impact on pain and concentration over time. High-dose subcutaneous BPG may be suitable for up to 3 months dosing intervals for secondary prophylaxis of RHD.
Madeira et al. 2016	Editorial
Mitchell et al. 2018	Qualitative study; assessing patients and clinicians experiences on BPG intramuscular pain.
Musoke et al. 2014	Qualitative study; Assessment of factors influencing adherence to secondary prophylaxis.
Oliveira et al. 2015	Wrong intervention; RCT assessing impact of alternative location for BPG injection on pain.
Russel et al. 2014	Not an RCT; Study assessing impact of choice of lidocaine, Buzzy, both or nothing on pain among patients receiving im BPG.
Saxena et al. 2015	Not an RCT: Observational study assessing adherence in a registry/tertiary center.
Sivri Bilgen et al. 2019	Wrong intervention; RCT assessing the effect of Buzzy or ShotBlocker on perceived pain in im injections.
Thomas et al. 2019	Wrong intervention; RCT assessing impact of needle temperature on pain among patients receiving penicillin injections
Tuğrul et al. 2014	Wrong intervention; RCT assessing impact of site and speed of injection on pain associated with im BPG.
Wyber et al. 2016	Qualitative study; consultation with global experts in RHD on the characteristics of BPG formulations which could be changed to improve adherence with secondary prophylaxis.
Zeydi et al. 2012	Letter to the Editor.

References

Adnan M, Tan KW, Lim B, Ng J, Rahiddeer M, Quek W. Comparison of patient pain experience: Benzathine penicillin 2.4 mega with water for injection vs benzathine penicillin 2.4 mega with lignocaine 1% for injection in patients with syphilis. Ann Acad Med Singap. 2013;42(Suppl.1):S311.

Basak T, Demirtas A, Yorubulut SM. Virtual reality and distraction cards to reduce pain during intramuscular benzathine penicillin injection procedure in adults: A randomised controlled trial. J Adv Nurs. 2021;77:2511-2518. doi: 10.1111/jan.14782.

Cooper J, Enkel SL, Moodley D, Dobinson H, Andersen E, Kado JH, Barr RK, Salman S, Baker MG, Carapetis JR, Manning L, Anderson A, Bennett J. "Hurts less, lasts longer" experiences of young people receiving high-dose subcutaneous infusions of benzathine penicillin G to prevent rheumatic heart disease. medRxiv 2023.09.13.23295467 doi: 10.1101/2023.09.13.23295467

Derya EY, Ukke K, Taner Y, Izzet AY. Applying Manual Pressure before Benzathine Penicillin Injection for Rheumatic Fever Prophylaxis Reduces Pain in Children. Pain Manag Nurs. 2015;16:328-35. doi: 10.1016/j.pmn.2014.08.013.

Douglas RM, Riley ID. Treatment of pneumonia in New Guinea. A controlled trial of crystalline penicillin and procaine penicillin aluminium monostearate. Med J Aust. 1971;1:1230-3.

Enkel SL, Kado J, Hla TK, Salman S, Bennett J, Anderson A, Carapetis JR, Manning L. Qualitative assessment of healthy volunteer experience receiving subcutaneous infusions of high-dose benzathine penicillin G (SCIP) provides insights into design of late phase clinical studies. PLoS One. 2023;18:e0285037. doi: 10.1371/journal.pone.0285037.

Farhadi A, Esmailzadeh M. Effect of local cold on intensity of pain due to Penicillin Benzathin intramuscular injection. International Journal of Medicine and Medical Sciences. 2011;3:343-345.

Feamley N, Brady S. Benzathine penicillin injections for the management of syphilis: One needle or two? Comparing patient perception, pain scores and cure rates of different injection techniques. Int J STD AIDS. 2020;31(Suppl12):78-79.

Hla TK, Salman S, Kado J, Moore BR, Manning L. Could late-latent syphilis be treated with a single subcutaneous infusion of long-acting penicillin? Sex Health. 2024:21:SH24003. doi: 10.1071/SH24003.

Huck DM, Nalubwama H, Longenecker CT, Frank SH, Okello E, Webel AR. A qualitative examination of secondary prophylaxis in rheumatic heart disease: factors influencing adherence to secondary prophylaxis in Uganda. Glob Heart. 2015;10:63-69.

Janier M, Libar E, Bonnet A, Meunier P, Tabet M, Mathourais M, Paterour C, Porcher R. Treatment of late syphilis with 2.4 million units benzathine penicillin G (BPG): tolerance of single versus divided doses. Sex Transm Dis. 2012;39:359-60. doi: 10.1097/OLQ.0b013e318249968c.

Jakhwal S. Assess effectiveness of Helfer Skin Tap on pain associated with intramuscular injection Benzathine pencillin in Rheumatic Heart disease patient. Clinical Trials Registry India. CTRI/2018/12/016745. Available at: https://trialsearch.who.int/Trial2.aspx?TrialID=CTRI/2018/12/016745

Kado JH, Salman S, Henderson R, Hand R, Wyber R, Page-Sharp M, Batty K, Carapetis J, Manning L. Subcutaneous administration of benzathine benzylpenicillin G has favourable pharmacokinetic characteristics for the prevention of rheumatic heart disease compared with intramuscular injection: a randomized, crossover, population pharmacokinetic study in healthy adult volunteers. J Antimicrob Chemother. 2020;75:2951-2959. doi: 10.1093/jac/dkaa282.

Kado J, Salman S, Hla TK, Enkel S, Henderson R, Hand RM, Hort A, Page-Sharp M, Batty K, Moore BR, Bennett J, Anderson A, Carapetis J, Manning L. Subcutaneous infusion of high-dose benzathine penicillin G is safe, tolerable, and suitable for less-frequent dosing for rheumatic heart disease secondary prophylaxis: a phase 1 open-label population pharmacokinetic study. Antimicrob Agents Chemother. 2023;67:e0096223. doi: 10.1128/aac.00962-23.

Madeira G, Mocumbi AO, Mayosi BM. Advice to health professionals: Use of lignocaine as a diluent to reduce the pain associated with the administration of benzathine penicillin G. S Afr Med J. 2016;106:742.

Mitchell AG, Belton S, Johnston V, Read C, Scrine C, Ralph AP. Aboriginal children and penicillin injections for rheumatic fever: how much of a problem is injection pain? Aust N Z J Public Health. 2018;42:46-51. doi: 10.1111/1753-6405.12737.

Musoke C, Mondo CK, Okello E, Zhang W, Kakande B, Nyakoojo W, Freers J. Benzathine penicillin adherence for secondary prophylaxis among patients affected with rheumatic heart disease attending Mulago Hospital. Cardiovasc J Afr. 2014;24:124-9.

Oliveira LF, Junqueira PS, Silva MR, Souza MM, Teles SA, Junqueira ALN. Ensaio clínico controlado randomizado: região ventro glútea, local alternativo para aplicação da benzilpenicilina benzatina G. Rev Eletronica Enferm. 2015;17(4):1-7.

Russell K, Nicholson R, Naidu R. Reducing the pain of intramuscular benzathine penicillin injections in the rheumatic fever population of Counties Manukau District Health Board. J Paediatr Child Health. 2014;50:112-7. doi: 10.1111/jpc.12400.

Saxena A, Mehta A, Ramakrishnan S. Adherence to benzathine penicillin in children with rheumatic fever/rheumatic heart disease: Results from an indian pediatric RHD registry. J Am Coll Cardiol. 2015;65(10 SUPPL.1): A2019.

Sivri Bilgen B, Balcı S. The Effect on Pain of Buzzy and ShotBlocker during the Administration of Intramuscular Injections to Children: A Randomized Controlled Trial. J Korean Acad Nurs. 2019;49:486-494. doi: 10.4040/jkan.2019.49.4.486.
Thomas N, Andrews R, Kaur S, Juneja R, Saxena A. Needle temperature and pain perception in the treatment of rheumatic heart disease. British Journal of Cardiac Nursing. 2019;14:134-138. doi: 10.12968/bjca.2019.14.3.134
Tuğrul E, Khorshıd L. Effect on pain intensity of injection sites and speed of injection associated with intramuscular penicillin.Int J Nurs Pract. 2014;20:468-74. doi: 10.1111/ijn.12161.
Wyber R, Boyd BJ, Colquhoun S, Currie BJ, Engel M, Kado J, Karthikeyan G, Sullivan M, Saxena A, Sheel M, Steer A, Mucumbitsi J, Zuhlke L, Carapetis J. Preliminary consultation on preferred product characteristics of benzathine penicillin G for secondary prophylaxis of rheumatic fever. Drug Deliv Transl Res. 2016;6(5):572-8.
Zeydi AE, Khezri HD. Can lidocaine be safely used to reduce pain caused by intramuscular penicillin injections? A short literature review. Oman Medical Journal. 2012;27:337.

Ongoing study

Pareeda T. A randomized controlled study of comparison of pain score after intramuscular injection of Benzathine penicillin in female patients with syphilis among cold compression before injection, 1% Lidocaine solution and combination between cold compression before injection and 1% Lidocaine solution. World Health Organization International Clinical Trials Registry Platform. Date of Registration: 3rd August 2020. TCTR20200803003 Available at: https://trialsearch.who.int/Trial2.aspx?TrialID=TCTR20200803003 , accessed 10th May 2024

Awaiting classification

Mariano AG. Pain Alleviation of Intramuscular Injection with Lidocaine versus Distilled Water in the Diluent of Benzathine Benzylpenicillin among Children with Rheumatic Fever and Rehumatic Heart Disease at MCU – FDTMF Hospital: A Prospective, Randomised Controlled Trial, Crossover Study. Master Thesis. 2023. Abstract available at: https://www.herdin.ph/index.php?view=research&cid=81593 , accessed 4th May 2024
Tabin C, Salvador ME, Manangan MR. Comparison of Pain Scale Using Lidocaine as a Diluent versus Lidocaine plus Coughing technique during Benzathine Penicillin G administration in Pediatric patients with Rheumatic Fever and Rheumatic Heart Disease. World Congress on Rheumatic Heart Disease 2023, 2-4 November 2023, Abu Dhabi; Abstract Id – 89 ; available at: https://world-heart-federation.org/world-congress-on-rhd/wp-content/uploads/sites/6/2023/10/WHF-Abstracts-52.pdf

Table S-2. Risk of Bias assessment

Study	Random Sequence Generation	Allocation Concealment	Blinding of Participants and Personnel	Blinding of Outcome Assessment	Incomplete Outcome Data	Selective Reporting	Other Bias
Amir 1998	Unclear Risk Judgement: No information provided	Unclear Risk Judgement: No information provided	Low Risk Judgement: "Only the attending nurses were aware of the group assignments"	Low Risk Judgement: "Only the attending nurses were aware of the group assignments"	Low Risk Judgement: Lost to FUP ≤5%	Unclear Risk Judgement: Protocol prior to publication not available	Unclear Risk Judgement: Mentions approval by Ethics Committee but provides no info on Trial Registration
Bycroft 2000	Low Risk Judgement: "The penicillin injections were randomised by a random number generator provided by SYSTAT as to which was given first and which was given second to each participant"	Unclear Risk Judgement: No information provided	Low Risk Judgement: "The injector and participant were blinded to the identify of which penicillin was being given. An attempt was made to blind the RN giving the injections. Each penicillin was drawn into a generic syringe by the co-investigator, hand warmed and wrapped in opaque tape. The RN giving the injections did not handle the loaded syringe until the injection was about to be given, therefore the RN was blinded until the injection was actually given. Due to the mechanics of giving the injection, the participant could not see the syringe during the injection nor the face or injection hand of the RN. This prevented the RN from giving nonverbal cues to the participant prior to or during the injection. Face to face	Low Risk Judgement: "The injector and participant were blinded to the identify of which penicillin was being given. An attempt was made to blind the RN giving the injections. Each penicillin was drawn into a generic syringe by the co-investigator, hand warmed and wrapped in opaque tape. The RN giving the injections did not handle the loaded syringe until the injection was about to be given, therefore the RN was blinded until the injection was actually given. Due to the mechanics of giving the injection, the participant could not see the syringe during the injection nor the face or injection hand of the RN. This prevented the RN from giving nonverbal cues to the participant prior to or during the injection. Face to face	Low Risk Judgement: Lost to FUP ≤5%	Unclear Risk Judgement: Protocol prior to publication not available. Methods section mentions observation for adverse reactions but these were not reported.	Unclear Risk Judgement: Mentions approval by Ethics Committee but provides no info on Trial Registration

			interaction between the RN and the participant after the injection was kept to a minimum.”	interaction between the RN and the participant after the injection was kept to a minimum.”			
Estrada 2019	Low Risk Judgement: “participants were randomly assigned to study groups by a computer generated randomised number system in blocks. The randomization was done 1:1:1:1 in blocks of four.”	Low Risk Judgement: “Randomization sequence was hidden for the investigators who made the selection of patients.”	Low Risk Judgement: “neither the patient nor the investigator who analyzed the pain results knew the assigned group.”	Low Risk Judgement: “neither the patient nor the investigator who analyzed the pain results knew the assigned group.”	Low Risk Judgement: Lost to FUP ≤5%	Unclear Risk Judgement: Reported primary and secondary outcomes as in the protocol. Protocol mentions adverse reactions but these were not reported – email sent to authors.	Low Risk Judgement: Registered trial (EudraCT 2014-003969-24)
Farhadi 2010	Unclear Risk Judgement: No information provided	Unclear Risk Judgement: No information provided	High Risk Judgement: No Blinding. Local cream was applied versus no placebo control.	High Risk: Judgement: No Blinding	Low Risk Judgement: Lost to FUP ≤5%	Low Risk Judgement: All planned outcomes were reported.	Low Risk Judgement: Registered trial (IRCT138904024247N1)
Harari 1988	Unclear Risk Judgement: No information provided	Unclear Risk Judgement: No information provided	Low Risk Judgement: Doctor and patient were unaware of which side received local anaesthetic.	Low Risk Judgement: Doctor and patient were unaware of which side received local anaesthetic.	High Risk Judgement: Less than 50% presented for assessment at 24h	Unclear Judgement: Protocol prior to publication not available	Unclear Risk Judgement: Mentions approval by Medical Research Advisory Committee but provides no info on Trial Registration
Jiamton 2022	Low Risk Judgement: “randomized using block randomization with a block size of four”	Unclear Risk Judgement: No information provided	Unclear Risk Judgement: Study described as double-blind and blinding mentioned for dermatologist who performs the injection and assesses patient, as well as for patients. However, methods for blinding are not described.	Unclear Risk Judgement: Study described as double-blind and blinding mentioned for dermatologist who performs the injection and assesses patient, as well as for patients. However, methods for blinding are not described.	Low Risk Judgement: Lost to FUP ≤5%	Low Risk Judgement: All planned outcomes were reported.	Low Risk Judgement: Registered trial (TCTR20180308001).
Morsy 2012	Unclear Risk Judgement: No information provided	Unclear Risk Judgement: No information provided	Low Risk Judgement: "The physician and patient were kept blind about the randomization. Only the 2 nurses in charge who were responsible for the	Low Risk Judgement: "The physician and patient were kept blind about the randomization. Only the 2 nurses in charge who were responsible for the	Low Risk Judgement: Lost to FUP ≤5%	Unclear Judgement: Protocol prior to publication not available	Unclear Risk Judgement: Mentions approval by Ethics Committee but provides no info on Trial Registration

			injection were aware about this randomization"	injection were aware about this randomization"			
Tamondong 2018	Low Risk<br>Judgement: “patients were randomly allocated into 2 groups using a computer generated randomization”	Unclear Risk<br>Judgement: No information provided	Low Risk<br>Judgement: “The physician, the patient and the nurse giving the medication were blinded on the patient randomization to either lidocaine or sterile water diluents"	Low Risk<br>Judgement: “The physician, the patient and the nurse giving the medication were blinded on the patient randomization to either lidocaine or sterile water diluents"	Low Risk<br>Judgement: Lost to FUP ≤5%	Unclear<br>Judgement: Protocol prior to publication not available	Unclear Risk<br>Judgement: Mentions approval by Institutional Review Board but provides no info on Trial Registration

Table S-3. Sub-analyses and Sensitivity analyses

Intramuscular Lidocaine versus Placebo				
Condition	Timing	Mean Difference	P value	I^2
Indication for Penicillin				
RHD/ARF [8, 10, 29]	Immediately after	-3.19 [-5.37 to -1.01]	0.004	96%
	After 2h/ 2 to 4h	-0.26 [-0.71 to 0.19]	0.26	0%
	24h	0.01 [-0.07 to 0.09]	0.84	0%
Syphilis [28]	Immediately after	-5.68 [-6.51 to -4.85]	<0.0001	NA
	5min after	-2.85 [-3.78 to -1.92]	<0.0001	NA
	20min after	-1.85 [-2.61 to -1.09]	<0.0001	NA
	24h	-0.33 [-1.21 to 0.55]	0.156	NA
GABS [24]	Immediately after	NA	0.0001	NA
	1h	NA	0.008	NA
	12h	NA	0.758	NA
Number of Centres				
Single-Centre [10, 28-29]	Immediately after	-4.74 [-5.84 to -3.63]	<0.0001	71%
	After 2h/ 2 to 4h	-1.00 [-2.63 to 0.63]	0.23	NA
	24h	0.09 [-0.34 to 0.51]	0.69	13%
Multicentre (two sites) [8]	Immediately after	-1.50 [-1.74 to -1.26]	<0.0001	NA
	After 2h	-0.20 [-0.67 to 0.27]	0.41	NA
	24h	0 [-0.08 to 0.08]	1.00	NA
Cross-over				
Cross-over [8, 10, 29]	Immediately after	-3.19 [-5.37 to -1.01]	0.004	96%
	After 2h/ 2 to 4h	-0.26 [-0.71 to 0.19]	0.26	0%
	24h	0.01 [-0.07 to 0.09]	0.84	0%
No Cross-Over [28]	Immediately after	-5.68 [-6.51 to -4.85]	<0.0001	NA
	5min after	-2.85 [-3.78 to -1.92]	<0.0001	NA
	20min after	-1.85 [-2.61 to -1.09]	<0.0001	NA
	24h	-0.33 [-1.21 to 0.55]	0.156	NA
