# Supplementary material for: Local anaesthetic to reduce injection pain in patients who are prescribed intramuscular benzathine penicillin G: a systematic review and meta-analysis
Source: eClinicalMedicine. 2024 Sep 4;76:102817. doi: 10.1016/j.eclinm.2024.102817 (PMC11404083; doi:10.1016/j.eclinm.2024.102817)
Supplement: Abstract Persian [file mmc2.docx]

*The following translations in Persian were submitted by the authors and we reproduce them as supplied. They have not been peer reviewed. Our editorial processes have only been applied to the original abstract in English, which should serve as reference for this manuscript*

**بی حسی موضعی برای کاهش درد تزریق بنزاتین پنیسیلین جی در بیماران: مرور سیستماتیک و فراتحلیل**

**چکیده**

**زمینه**: تزریق بنزاتین پنیسیلین جی (بی پی جی) هر سه یا چهار هفته برای دوره ای طولانی (مانند 10 سال، تا 40 سالگی، یا تمام عمر) برای پیشگیری از عفونت های استرپتوکوکی گروه آ که به تب روماتیسمی حاد مکرر با پتانسیل پیشرفت به بیماری روماتیسم قلبی می انجامند ، توصیه شده است. مدت درمان و فراوانی تکرار درمان و درد مربوط به تزریق ممکن است منجر به کاهش پذیرش درمان در میان بیماران شود و دوره های کوتاهتر درمان برای سیفلیس و عفونت های استرپتوکوکی توصیه شده است. این پژوهش اثرات بی حسی موضعی در کاهش درد تزریق بی پی جی را بررسی می کند.

**روش ها**: در این پژوهش، سنترال، مدلاین، امبیس، نمایه استنادی چکیده کنفرانس های علمی، لایلکس و متون خاکستری از آغاز تا چهارم می 2024 جستجو شد. کارآزمایی های بالینی تصادفی شده با گروه کنترل که بی پی جی را با بی پی جی در کنار بی حسی موضعی (با هر اندیکاسیون و هرگونه بی حسی موضعی برای تسکین درد) مقایسه می کنند ، وارد این بررسی شدند. ما روش GRADE را برای گزارش درجه اطمینان به کار بردیم. داده های خلاصه شده از کارآزمایی ها گردآوری و پیامدهای اولیه (درد تزریق) از طریق تفاضل میانگین بررسی شدند. به دلیل ناهمگنی پژوهش ها، مدل تاثیرات-تصادفی برای تحلیل به کار رفت. پروتکل این پژوهش در پروسپرو به شماره CRD42022342437 ثبت شد.

**یافته ها**: از جستجو 3958 رکورد به علاوه 3 رکورد از طریق متون جستجوی متون خاکستری به دست آمد. پس از حذف رکوردهای تکراری و غربالگری چکیده ها و متن کامل، هشت کارآزمایی با 489 بیمار (151 با بیماری روماتیسم قلبی) وارد مطالعه شدند. بیماران در بیشتر مطالعات، سطح درد آنی را بسیار شدید و در 24 ساعت بعدی با شدت کمتر گزارش کرده بودند. کاربرد لیدوکائین همراه بی پی جی به کاهش قابل توجه درد آنی پس از تزریق (تفاضل میانگین -3.84، فاصله اطمینان 95% -6.19 تا -1.48، p=0.0001؛ چهار مطالعه؛ I^2^=98%؛ GRADE: متوسط)، درد در پنج دقیقه پس از تزریق (تفاضل میانگین -2.85، فاصله اطمینان 95% -3.78 تا -1.92، p<0.0001 ؛ یک مطالعه؛ GRADE: متوسط)، و درد در 20 دقیقه پس از تزریق (تفاضل میانگین -1.85، فاصله اطمینان 95% -2.61 تا -1.09، p<0.0001 ؛ یک مطالعه؛ GRADE: متوسط) بر مبنای نمره 1 تا 10 برای درد انجامید. تنها مطالعه ای که به بررسی مالیدن کرم لیدوکائین پیش از تزریق بر روی پوست محل تزریق پرداخته بود، کاهش درد قابل توجهی را نشان نداد (تفاضل میانگین -0.54، فاصله اطمینان 95% 1.17 تا 0.09، p=0.13 ؛ یک مطالعه؛ GRADE: پایین). ترکیب مپیواکائین با بی پی جی در بیماران مبتلا به سیفلیس، کاهش درد آنی قابل توجهی را نشان داد (تفاضل میانگین -2.19، فاصله اطمینان 95% -2.49 تا -1.89، p<0.0001 ؛ یک مطالعه؛ GRADE: متوسط). دو مطالعه با بررسی ترکیب پروکائین با بی پی جی، سطح درد آنی یا درد یک ساعت پس از تزریق کمتری (تفاضل میانگین و فاصله اطمینان ارائه نشده است؛ به ترتیب p=0.001 و p=0.008؛ یک مطالعه؛ GRADE: پایین)، یا درد آنی یا درد 24 ساعت پس از تزریق (در ماهیچه باسن) کمتری (تفاضل میانگین و فاصله اطمینان ارائه نشده است؛ برای هر دو p<0.001؛ یک مطالعه؛ GRADE: پایین) را گزارش کردند. عوارض جانبی شدیدی در مطالعات گزارش نشده است.

**تفسیر**: در بیمارانی که تزریق ماهیچه ای بی پی جی دریافت می کنند، شواهد آماری با درجه اطمینان متوسط پیشنهاد می کنند که تزریق با لیدوکائین یا مپیواکائین در مقایسه با تزریق بی پی جی با آب قابل تزریق، ممکن است میزان درد پس از تزریق را بهبود بخشد. پروکائین هم شاید سودمند باشد ولی درجه اطمینان شواهد پایین است. بیشتر پژوهش های این بررسی دارای حجم نمونه ای کوچک از بیماران بودند و میزان درد را در زمان های متفاوتی اندازه گرفته بودند. به خاطر داده های ناکافی، ما نتوانستیم تاثیر حجم تزریق و دوز بی حس کننده های موضعی را بر شدت درد و مدت زمان تسکین درد بررسی کنیم.

**تامین کننده بودجه این پژوهش**: سازمان جهانی بهداشت (WHO)

**کلیدواژگان**: لیدوکائین، بیماری روماتیسم قلبی، سیفلیس، ایمپتیگو (زردزخم)، فارنژیت استرپتوکوکی
