# Supplementary material for: Local anaesthetic to reduce injection pain in patients who are prescribed intramuscular benzathine penicillin G: a systematic review and meta-analysis
Source: eClinicalMedicine. 2024 Sep 4;76:102817. doi: 10.1016/j.eclinm.2024.102817 (PMC11404083; doi:10.1016/j.eclinm.2024.102817)
Supplement: Abstract Urdu [file mmc3.docx]

*The following translations in Urdu were submitted by the authors and we reproduce them as supplied. They have not been peer reviewed. Our editorial processes have only been applied to the original abstract in English, which should serve as reference for this manuscript*

مقامی بے ہوشی کی دوا کے ذریعے انجکشن کے درد کو کم کرنا ان مریضوں میں جنہیں بینزاتھین پینسلن جی کے انٹرامسکلر انجکشن تجویز کیے گئے ہیں: ایک منظم جائزہ اور میٹا تجزیہ

خلاصہ

پس منظر: تین سے چار ہفتہ وار بینزاتھین پینسلن جی (BPG) کے انٹرامسکلر انجکشن طویل مدت کے لئے (مثلاً 10 سال، 40 سال کی عمر تک، یا زندگی بھر) تجویز کیے جاتے ہیں تاکہ گروپ اے اسٹریپٹوکوکل انفیکشن کو روکا جا سکے جو کہ بار بار ہونے والے ایکیوٹ ریمیٹک فیور (ARF) اور ریمیٹک دل کی بیماری (RHD) کی طرف بڑھنے کا سبب بنتے ہیں۔ علاج کی مدت، بار بار انجکشن کی ضرورت اور BPG انجکشن کے ساتھ جڑے مقامی درد کی وجہ سے مریضوں کی پیروی کم ہو سکتی ہے۔ سفلس اور اسٹریپٹوکوکل انفیکشن کے علاج کے لئے BPG کے مختصر دورانیے کے کورس تجویز کیے جاتے ہیں۔ ہم نے ان مریضوں میں مقامی بے ہوشی کی دوا کے ذریعے انجکشن کے درد کو کم کرنے کے اثرات کا جائزہ لینے کا ارادہ کیا جو BPG کے ساتھ علاج کر رہے ہیں۔

طریقے: اس منظم جائزہ اور میٹا تجزیے میں، ہم نے کوکرین سنٹرل رجسٹر آف کنٹرولڈ ٹرائلز، میڈلائن، ایمبیس، کانفرنس پروسیڈنگز سائٹیشن انڈیکس-سائنس اور لیلاکس میں ڈیٹا بیس کی ابتدا سے لے کر 4 مئی 2024 تک تلاش کی اور مزید تلاشیں گرے لٹریچر کے لئے کیں۔ وہ رینڈمائزڈ کنٹرولڈ ٹرائلز شامل کیے گئے جو BPG کو مقامی بے ہوشی کی دواؤں کے ساتھ استعمال کرنے کا موازنہ کرتے تھے۔ ہم نے GRADE کو ثبوت کے معیار کی جانچ کے لئے لاگو کیا۔ شامل شدہ ٹرائلز سے خلاصہ ڈیٹا نکالا گیا۔ بنیادی نتیجہ انجکشن کے درد کا تھا، جو کہ اوسط فرق کے ذریعے جانچا گیا۔ مطالعے کی مختلفیت کو مدنظر رکھتے ہوئے رینڈم ایفیکٹس ماڈل استعمال کیا گیا۔ یہ مطالعہ پروسپرو میں رجسٹرڈ ہے، CRD42022342437۔

نتائج: ڈیٹا بیس کی تلاشوں میں کل 3,958 ریکارڈز شناخت ہوئے، اور 3 اضافی ریکارڈز گرے لٹریچر کی تلاشوں سے حاصل کیے گئے۔ نقلیں ہٹانے، خلاصوں کی جانچ اور مکمل متن کے جائزے کے بعد، آٹھ ٹرائلز شامل کیے گئے، جن میں کل 489 مریض (151 مریض RHD کے ساتھ) شامل تھے۔ مریضوں کی رپورٹ کردہ فوری درد کی سطح زیادہ شدت کی تھی۔ 24 گھنٹوں بعد بھی کم شدت کا درد رپورٹ کیا گیا۔ BPG کے ساتھ لڈوکین کے ملانے سے فوری بعد انجکشن کے درد میں نمایاں کمی دیکھی گئی (اوسط فرق -3.84، 95% اعتماد کے وقفہ -6.19 سے -1.48، P=0.0001؛ 4 مطالعے؛ I2=98%; GRADE: درمیانی معیار)، 5 منٹ بعد درد (اوسط فرق -2.85، 95% اعتماد کا وقفہ -3.78 سے -1.92، P<0.0001؛ 1 مطالعہ؛ GRADE: درمیانی معیار)، اور 20 منٹ بعد درد (اوسط فرق -1.85، 95% اعتماد کا وقفہ -2.61 سے -1.09، P<0.0001؛ 1 مطالعہ؛ GRADE: درمیانی معیار) 1 سے 10 پیمانے پر۔ ایک مطالعے میں BPG انجکشن سے پہلے جلد پر لگائی جانے والی لڈوکین کریم کی جانچ کی گئی اور انجکشن کے درد میں کوئی نمایاں کمی نہیں پائی گئی (اوسط فرق= -0.54، 95% اعتماد کا وقفہ -1.17 سے 0.09، P=0.13؛ 1 مطالعہ؛ GRADE: کم معیار)۔ سفلس کے مریضوں میں BPG کے ساتھ میپیواکین کے ملانے سے فوری بعد انجکشن کے درد میں نمایاں کمی دیکھی گئی (اوسط فرق -2.19، 95% اعتماد کا وقفہ -2.49 سے -1.89، P<0.0001؛ 1 مطالعہ؛ GRADE: درمیانی معیار)۔ دو مطالعے BPG کے ساتھ پروکین کے ملانے کی جانچ کرتے ہوئے رپورٹ کیے گئے: فوری درد کی سطح یا 1 گھنٹہ بعد درد میں کمی (اوسط فرق اور 95% اعتماد کے وقفے فراہم نہیں کیے گئے، P=0.001 اور P=0.008، بالترتیب؛ 1 مطالعہ؛ GRADE: کم معیار)، یا کم فوری درد اور 24 گھنٹوں بعد درد پروکین کے ساتھ ملائے گئے BPG کے ساتھ انجکشن کے بعد (اوسط فرق اور 95% اعتماد کے وقفے فراہم نہیں کیے گئے، P<0.001 دونوں کے لئے؛ 1 مطالعہ؛ GRADE: کم معیار)۔ کوئی شدید منفی ردعمل رپورٹ نہیں ہوا۔

تشریح: ان مریضوں میں جو انٹرامسکلر BPG انجکشن وصول کر رہے ہیں، درمیانی معیار کے مقداری ثبوت سے پتہ چلتا ہے کہ BPG کے ساتھ ملائے گئے لڈوکین یا میپیواکین کے انجکشن انجکشن کے بعد کے درد کے سکور کو بہتر بنا سکتے ہیں جیسا کہ BPG کے ساتھ ملائے گئے سٹرل پانی کے مقابلے میں۔ پروکین بھی فائدہ مند ہو سکتا ہے، لیکن ثبوت کا معیار کم تھا۔ زیادہ تر مطالعات میں چھوٹے مریض نمونے شامل تھے اور مختلف وقتوں پر درد کی سطحوں کا اندازہ لگایا گیا تھا۔ ناکافی ڈیٹا کی وجہ سے ہم انجکشن کے حجم اور مقامی بے ہوشی کی دواؤں کی خوراک کے اثرات کا اندازہ نہیں لگا سکے۔

فنڈنگ: عالمی ادارہ صحت

کلیدی الفاظ:** لڈوکین؛ ریمیٹک؛ سفلس؛ امپیٹیگو؛ اسٹریپٹوکوکل فارینجائٹس۔
