# Supplementary material for: Local anaesthetic to reduce injection pain in patients who are prescribed intramuscular benzathine penicillin G: a systematic review and meta-analysis
Source: eClinicalMedicine. 2024 Sep 4;76:102817. doi: 10.1016/j.eclinm.2024.102817 (PMC11404083; doi:10.1016/j.eclinm.2024.102817)
Supplement: Abstract French [file mmc4.docx]

*The following translations in French were submitted by the authors and we reproduce them as supplied. They have not been peer reviewed. Our editorial processes have only been applied to the original abstract in English, which should serve as reference for this manuscript*

**Anesthésie locale pour réduire la douleur des injections de pénicilline G benzathine intramusculaire : une revue systématique et une méta-analyse**

**Résumé**

**Contexte** : Les injections intramusculaires de pénicilline G benzathine (BPG) toutes les 3 à 4 semaines pendant une période prolongée (par exemple, durée de 10 ans, jusqu’à l’âge de 40 ans, ou à vie) sont recommandées pour prévenir les infections à streptocoques du groupe A, responsables des récidives de rhumatisme articulaire aigu (RAA) et de la progression potentielle vers une cardiopathie rhumatismale (CR). La durée du traitement, la fréquence et la douleur locale associée aux injections de BPG peuvent conduire à une mauvaise adhérence au traitement. Des traitements plus courts de BPG sont recommandés pour le traitement de la syphilis et des infections à streptocoques. Nous avons cherché à évaluer les effets de l'anesthésie locale pour réduire la douleur des injections chez les patients traités avec de la BPG au long cours.

**Méthodes** : Dans cette revue systématique et méta-analyse, nous avons recherché dans le Cochrane Central Register of Controlled Trials, MEDLINE, EMBASE, Conference Proceedings Citation Index‐Science et LILACS depuis le début des bases de données jusqu’au 4 mai 2024, et avons effectué des recherches supplémentaires pour la littérature grise. Les essais contrôlés randomisés comparant la BPG versus la BPG administrée avec des anesthésiques locaux ont été inclus. Les essais contrôlés randomisés utilisant la BPG, indépendamment de l'indication, et testant tout agent anesthésique local pour l'atténuation de la douleur ont été considérés comme éligibles. Nous avons appliqué GRADE pour évaluer la qualité des preuves. Les données résumées ont été extraites des essais inclus. L’issue principale était la douleur des injections, évaluée par les différences moyennes. Un modèle à effets aléatoires a été utilisé pour tenir compte de l'hétérogénéité des études. Cette étude est enregistrée auprès de PROSPERO, CRD42022342437.

**Résultats** : Les recherches dans les bases de données ont identifié un total de 3 958 enregistrements, et 3 enregistrements supplémentaires ont été identifiés à partir des recherches de littérature grise. Après élimination des doublons, sélection des résumés et revue des textes complets, huit essais ont été inclus, combinant un total de 489 patients (151 patients avec une CR). Le niveau de douleur immédiate, tel que rapporté par les patients, était de haute intensité dans la plupart des études. Une douleur de faible intensité a été signalée après 24 heures. L’administration de lidocaïne mélangée à la BPG était associée à une réduction significative de la douleur immédiate post-injection (différence moyenne -3,84, intervalle de confiance à 95 % -6,19 à -1,48, P=0,0001 ; 4 études ; I2=98 % ; GRADE : qualité modérée), douleur à 5 minutes (différence moyenne -2,85, intervalle de confiance à 95 % -3,78 à -1,92, P<0,0001 ; 1 étude ; GRADE : qualité modérée), et douleur à 20 minutes (différence moyenne -1,85, intervalle de confiance à 95 % -2,61 à -1,09, P<0,0001 ; 1 étude ; GRADE : qualité modérée) sur une échelle de 1 à 10. Une étude a évalué la crème de lidocaïne appliquée sur la peau avant l'injection de BPG et n'a montré aucune réduction significative de la douleur d'injection (différence moyenne = -0,54, intervalle de confiance à 95 % -1,17 à 0,09, P=0,13 ; 1 étude ; GRADE : qualité faible). La mépivacaïne mélangée à la BPG chez les patients atteints de syphilis a montré une réduction significative de la douleur immédiate post-injection (différence moyenne -2,19, intervalle de confiance à 95 % -2,49 à -1,89, P<0,0001 ; 1 étude ; GRADE : qualité modérée). Deux études ont évalué la procaïne mélangée à la BPG et ont rapporté : des niveaux de douleur immédiate plus faibles ou une douleur évaluée à 1 heure (les différences moyennes et les intervalles de confiance à 95 % ne sont pas fournis, P=0,001 et P=0,008, respectivement ; 1 étude ; GRADE : qualité faible), ou moins de douleur immédiate et douleur à 24 heures sur la fesse injectée avec la procaïne mélangée à la BPG (les différences moyennes et les intervalles de confiance à 95 % ne sont pas fournis, P<0,001 pour les deux ; 1 étude ; GRADE : qualité faible). Aucune réaction indésirable sévère n’a été rapportée.

**Interprétation** : Chez les patients recevant des injections intramusculaires de BPG, des preuves quantitatives de qualité modérée suggèrent que les injections de BPG diluées avec de la lidocaïne ou de la mépivacaïne peuvent améliorer les scores de douleur post-injection par rapport aux injections de BPG diluées avec de l'eau stérile. La procaïne peut également présenter un avantage, mais la qualité des preuves était inférieure. La majorité des études incluaient des petits échantillons de patients et ont évalué les niveaux de douleur à différents moments. En raison de données insuffisantes, nous n'avons pas pu évaluer l'impact du volume d'injection et de la dose des anesthésiques locaux sur l'intensité de la douleur et la durée du soulagement de la douleur.

**Financement** : OMS

**Mots-clés** : lidocaïne ; rhumatismale ; syphilis ; impétigo ; pharyngite à streptocoque.
