# Supplementary material for: Local anaesthetic to reduce injection pain in patients who are prescribed intramuscular benzathine penicillin G: a systematic review and meta-analysis
Source: eClinicalMedicine. 2024 Sep 4;76:102817. doi: 10.1016/j.eclinm.2024.102817 (PMC11404083; doi:10.1016/j.eclinm.2024.102817)
Supplement: Abstract in Spanish [file mmc5.docx]

*The following translations in Spanish were submitted by the authors and we reproduce them as supplied. They have not been peer reviewed. Our editorial processes have only been applied to the original abstract in English, which should serve as reference for this manuscript*

**Anestésico local para reducir el dolor de inyección en pacientes a los que se les prescribe penicilina G benzatina intramuscular: una revisión sistemática y un meta-análisis**

**Resumen**

**Antecedentes:** Las inyecciones intramusculares de penicilina G benzatina (BPG) cada 3 o 4 semanas son esenciales para prevenir las infecciones estreptocócicas del grupo A, la fiebre reumática recurrente y la enfermedad reumática del corazón. El dolor asociado con estas inyecciones frecuentes a menudo lleva a una menor adherencia, especialmente durante períodos prolongados de tratamiento. Esta revisión sistemática y meta-análisis tuvo como objetivo evaluar la efectividad de los anestésicos locales en la reducción del dolor de inyección en pacientes tratados con BPG.

**Métodos:** La revisión incluyó ensayos controlados aleatorizados que compararon inyecciones de BPG solas con inyecciones de BPG con anestésicos locales. Se buscaron fuentes como el Registro Central Cochrane de Ensayos Controlados, MEDLINE, EMBASE, el Índice de Citas de Procedimientos de Conferencias de Ciencias y LILACS hasta el 4 de mayo de 2024. También se realizaron búsquedas adicionales en literatura gris. El *endpoint* principal fue el dolor de inyección, evaluado a través de diferencias medias, utilizando un modelo de efectos aleatorios para considerar la heterogeneidad del estudio. Se aplicó GRADE para evaluar la calidad de la evidencia. El estudio está registrado en PROSPERO (CRD42022342437).

**Hallazgos:** Las búsquedas en bases de datos identificaron un total de 3,958 registros, y se recuperaron 3 registros adicionales de búsquedas en literatura gris. Después de la eliminación de duplicados, la evaluación de resúmenes y la revisión de textos completos, se incluyeron ocho ensayos, combinando un total de 489 pacientes (151 pacientes con enfermedad reumática del corazón). El nivel de dolor inmediato, según lo informado por los pacientes, fue de alta intensidad en la mayoría de los estudios. Se informó dolor de baja intensidad a las 24 horas. La administración de lidocaína mezclada con BPG se asoció con una reducción significativa en el dolor inmediato post-inyección (diferencia media -3.84, intervalo de confianza del 95% -6.19 a -1.48, P=0.0001; 4 estudios; I2=98%; GRADE: calidad moderada), dolor a los 5 minutos (diferencia media -2.85, intervalo de confianza del 95% -3.78 a -1.92, P<0.0001; 1 estudio; GRADE: calidad moderada) y dolor a los 20 minutos (diferencia media -1.85, intervalo de confianza del 95% -2.61 a -1.09, P<0.0001; 1 estudio; GRADE: calidad moderada) en una escala del 1 al 10. Un estudio evaluó la crema de lidocaína aplicada en la piel antes de la inyección de BPG y no mostró una reducción significativa en el dolor de inyección (diferencia media = -0.54, intervalo de confianza del 95% -1.17 a 0.09, P=0.13; 1 estudio; GRADE: calidad baja). La mepivacaína mezclada con BPG en pacientes con sífilis mostró una reducción significativa del dolor inmediato post-inyección (diferencia media -2.19, intervalo de confianza del 95% -2.49 a -1.89, P<0.0001; 1 estudio; GRADE: calidad moderada). Dos estudios evaluaron la procaína mezclada con BPG y reportaron: niveles de dolor inmediato más bajos o dolor evaluado a la 1 hora (diferencia media e intervalos de confianza del 95% no proporcionados, P=0.001 y P=0.008, respectivamente; 1 estudio; GRADE: calidad baja), o menos dolor inmediato y dolor a las 24 horas en la nalga inyectada con procaína mezclada con BPG (diferencia media e intervalos de confianza del 95% no proporcionados, P<0.001 para ambos; 1 estudio; GRADE: calidad baja). No se informaron reacciones adversas graves.

**Interpretación**

En pacientes tratados con inyecciones intramusculares de BPG, la evidencia cuantitativa de calidad moderada sugiere que la dilución de BPG con lidocaína o mepivacaína puede mejorar los niveles de dolor post-inyección en comparación con las inyecciones de BPG diluidas con agua estéril. La procaína también puede tener un beneficio, pero la calidad de la evidencia es menor. La mayoría de los estudios incluyeron pequeñas muestras de pacientes y evaluaron los niveles de dolor en diferentes momentos. Debido a datos insuficientes, no pudimos evaluar el impacto del volumen de la inyección y de la dosis de los anestésicos locales en la intensidad del dolor y la duración del alivio del dolor.

**Financiación**

Este estudio fue financiado por la Organización Mundial de la Salud (OMS).

**Palabras Clave**

Lidocaína, reumática, sífilis, impétigo, faringitis estreptocócica.
