# Supplementary material for: Local anaesthetic to reduce injection pain in patients who are prescribed intramuscular benzathine penicillin G: a systematic review and meta-analysis
Source: eClinicalMedicine. 2024 Sep 4;76:102817. doi: 10.1016/j.eclinm.2024.102817 (PMC11404083; doi:10.1016/j.eclinm.2024.102817)
Supplement: Abstract portugues [file mmc6.docx]

*The following translations in Portuguese were submitted by the authors and we reproduce them as supplied. They have not been peer reviewed. Our editorial processes have only been applied to the original abstract in English, which should serve as reference for this manuscript*

**Anestesia local para reduzir a dor da injeção em doentes tratados com penicilina G benzatina intramuscular: uma revisão sistemática e meta-análise**

**Resumo**

**Antecedentes**: Injeções intramusculares de penicilina G benzatina (BPG) a cada 3 a 4 semanas por um período prolongado (por exemplo, 10 anos, até os 40 anos de idade ou por toda a vida) são recomendadas para prevenir infeções estreptocócicas do grupo A que causam febre reumática aguda (FRA) recorrente e potencial progressão cardiopatia reumática (CR). A duração do tratamento, a frequência e a dor local associada às injeções de BPG podem levar a uma redução na adesão ao tratamento. Cursos mais curtos de BPG são recomendados para o tratamento da sífilis e infeções estreptocócicas. O nosso objetivo foi avaliar os efeitos da anestesia local na redução da dor da injeção em doentes tratados com BPG.

**Métodos**: Nesta revisão sistemática e meta-análise, pesquisámos o *Cochrane Central Register of Controlled Trials*, MEDLINE, EMBASE, C*onference Proceedings Citation Index-Science* e LILACS desde o início das bases de dados até 4 de maio de 2024, e realizámos buscas adicionais de literatura cinzenta. Ensaios clínicos randomizados comparando BPG versus BPG administrada juntamente com anestésicos locais foram incluídos. Ensaios utilizando BPG, independentemente da indicação, e testando qualquer agente anestésico local para alívio da dor foram considerados elegíveis. Aplicámos o GRADE para avaliar a qualidade da evidência. Dados foram extraídos dos ensaios incluídos. O *endpoint* primário foi a dor da injeção, avaliada por meio de diferenças médias. Um modelo de *random-effects* foi utilizado considerando a heterogeneidade dos estudos. Esta revisão está registada no PROSPERO, CRD42022342437.

**Resultados**: As buscas nas bases de dados identificaram um total de 3.958 registos, e 3 registos adicionais foram recuperados em buscas na literatura cinzenta. Após a remoção de duplicados, avaliação de resumos e revisão de textos completos, oito ensaios foram incluídos, combinando um total de 489 pacientes (151 pacientes com DCR). O nível de dor imediata, conforme relatado pelos pacientes, foi de alta intensidade na maioria dos estudos. Dor de baixa intensidade foi relatada após 24h. A administração de lidocaína misturada com BPG foi associada a uma redução significativa na dor imediata pós-injeção (diferença média -3,84, intervalo de confiança de 95% -6,19 a -1,48, P=0,0001; 4 estudos; I2=98%; GRADE: qualidade moderada), dor aos 5min (diferença média -2,85, intervalo de confiança de 95% -3,78 a -1,92, P<0,0001; 1 estudo; GRADE: qualidade moderada), e dor aos 20min (diferença média -1,85, intervalo de confiança de 95% -2,61 a -1,09, P<0,0001; 1 estudo; GRADE: qualidade moderada) em uma escala de 1 a 10. Um estudo avaliou o creme de lidocaína aplicado na pele antes da injeção de BPG e não mostrou redução significativa na dor da injeção (diferença média = -0,54, intervalo de confiança de 95% -1,17 a 0,09, P=0,13; 1 estudo; GRADE: qualidade baixa). A mepivacaína misturada com BPG em pacientes com sífilis mostrou uma redução significativa da dor imediata pós-injeção (diferença média -2,19, intervalo de confiança de 95% -2,49 a -1,89, P<0,0001; 1 estudo; GRADE: qualidade moderada). Dois estudos avaliaram a procaína misturada com BPG e relataram: níveis de dor imediata mais baixos ou dor avaliada após 1h (diferença média e intervalos de confiança de 95% não fornecidos, P=0,001 e P=0,008, respetivamente; 1 estudo; GRADE: qualidade baixa), ou menos dor imediata e dor após 24h na nádega injetada com procaína misturada com BPG (diferença média e intervalos de confiança de 95% não fornecidos, P<0,001 para ambos; 1 estudo; GRADE: qualidade baixa). Nenhuma reação adversa grave foi relatada.

**Interpretação**: Em doentes tratados com injeções intramusculares de BPG, evidência quantitativa de qualidade moderada sugere que diluição de BPG com lidocaína ou mepivacaína pode melhorar os níveis de dor pós-injeção em comparação com injeções de BPG diluídas com água estéril. A procaína também pode ter um benefício, mas a qualidade da evidência é menor. A maioria dos estudos incluiu pequenas amostras de pacientes e avaliou os níveis de dor em diferentes momentos. Devido a dados insuficientes, não pudemos avaliar o impacto do volume da injeção e da dose dos anestésicos locais na intensidade da dor e na duração do alívio da dor.

**Financiamento**: OMS

**Palavras-chave**: lidocaína; reumática; sífilis; impetigo; faringite estreptocócica.
